# Site-specific incorporation of citrulline into proteins in mammalian cells

**DOI:** 10.1038/s41467-020-20279-w

**Published:** 2021-01-04

**Authors:** Santanu Mondal, Shu Wang, Yunan Zheng, Sudeshna Sen, Abhishek Chatterjee, Paul R. Thompson

**Affiliations:** 1 Department of Biochemistry and Molecular Pharmacology, UMass Medical School, 364 Plantation Street, Worcester, MA 01605 USA; 2 Department of Chemistry, Boston College, Chestnut Hill, MA 02467 USA

**Keywords:** Synthetic biology, Chemical genetics, Proteomics, Hydrolases

## Abstract

Citrullination is a post-translational modification (PTM) of arginine that is crucial for several physiological processes, including gene regulation and neutrophil extracellular trap formation. Despite recent advances, studies of protein citrullination remain challenging due to the difficulty of accessing proteins homogeneously citrullinated at a specific site. Herein, we report a technology that enables the site-specific incorporation of citrulline (Cit) into proteins in mammalian cells. This approach exploits an engineered *E. coli*-derived leucyl tRNA synthetase-tRNA pair that incorporates a photocaged-citrulline (SM60) into proteins in response to a nonsense codon. Subsequently, SM60 is readily converted to Cit with light in vitro and in living cells. To demonstrate the utility of the method, we biochemically characterize the effect of incorporating Cit at two known autocitrullination sites in Protein Arginine Deiminase 4 (PAD4, R372 and R374) and show that the R372Cit and R374Cit mutants are 181- and 9-fold less active than the wild-type enzyme. This technology possesses the potential to decipher the biology of citrullination.

## Abstract (web summary)

Citrullination of arginine is crucial for several physiological processes. Here the authors report the site-specific incorporation of citrulline into proteins in mammalian cells using an engineered tRNA synthetase/tRNA pair and a photocaged-citrulline.

## Introduction

**Fig. 1:**
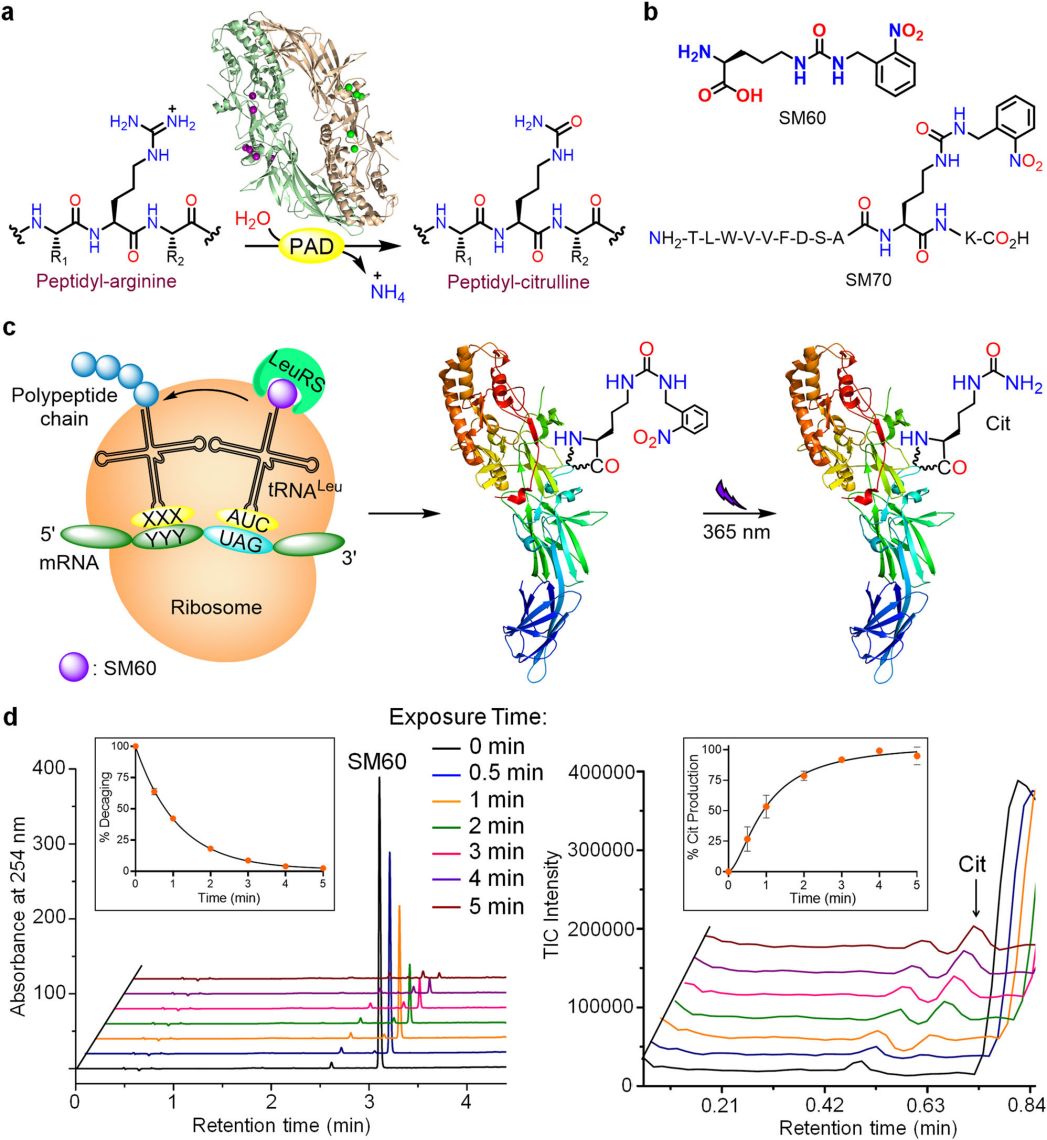
SM60, a photocaged-citrulline and its conversion to citrulline with 365 nm UV light. **a** Conversion of peptidyl-arginine to peptidyl-citrulline by the PADs. **b** Chemical structures of SM60 and SM70. **c** Schematic representation of the incorporation of SM60 into proteins by an engineered LeuRS-tRNA^Leu^ pair and subsequent conversion to citrulline. **d** Decaging of SM60 to citrulline with 365 nm UV irradiation. Left and right panels indicate the HPLC and ion chromatograms, showing the disappearance of SM60 and the formation of citrulline (Cit), respectively, with increasing UV exposure. Quantitative analyses of decaging and Cit formation are shown in the insets. *n* = 2 independent experiments, data are presented as mean value ± SD. Assay mixture: 1 mM SM60, 2 mM DTT, phosphate-buffered saline pH 7.4. Source data are provided as a Source Data file.

Citrullination is a post-translational modification (PTM) that involves the hydrolysis of the positively charged guanidium group on arginine to generate a neutral urea (Fig. 1a)^1^. Citrullination plays crucial roles in many physiological processes, including the epigenetic regulation of gene transcription, neutrophil extracellular trap (NET) formation or NETosis, and maintaining pluripotency^1–7^. Citrullination is catalyzed by the protein arginine deiminases (PADs) (Fig. 1a), a group of four catalytically active cysteine hydrolases (PAD1–4)^8^. PADs are Ca^2+^-dependent enzymes and the presence of calcium increases PAD activity by >10,000-fold. Calcium-binding leads to dramatic conformational rearrangements, particularly of the nucleophilic cysteine (C645 in PAD1, 4; C647 in PAD2; C646 in PAD3) to form a catalytically competent active site^9^.

Aberrant protein citrullination is a hallmark of multiple autoimmune disorders, including rheumatoid arthritis (RA), multiple sclerosis (MS), ulcerative colitis (UC), and lupus, as well as several neurodegenerative diseases and cancer. Of note, multiple pan- and isozyme-selective PAD inhibitors are known and these inhibitors show efficacy in animal models of RA, UC, MS, lupus, and sepsis^1,8,10–12^. The contribution of protein hypercitrullination to the pathology of various diseases has been further established using the phenylglyoxal (PG)-based citrulline (Cit)-specific probes, Rhodamine-PG (Rh-PG) and Biotin-PG^1,8^. For example, Rh-PG enabled visualization of extensive citrullination of serum proteins and a marked decrease upon treatment with pan-PAD inhibitor, Cl-amidine, in a mouse model of UC^13^. Using Biotin-PG and a chemoproteomic platform, we also identified various classes of novel citrullinated proteins, including serine protease inhibitors (SERPINs), serine proteases, transport proteins, and complement system components along with known citrullinated proteins (e.g., vimentin, enolase, keratin, and fibrin) in the serum, synovial fluid, and synovial tissue of RA patients^14^. Although the list of citrullinated proteins is ever-expanding, the effect of citrullination on the structure and activity of a given protein remains poorly understood.

The most commonly used strategy for generating a citrullinated protein involves its treatment with a PAD. However, this leads to citrullination at all sites that are available in vitro, which may not fully recapitulate the situation in vivo. Moreover, the degree of modification at each site is frequently partial, leading to a complex heterogeneous mixture^14–16^. Clearly, this strategy fails to provide information on the effect of individual citrullination events, underscoring the need for a method to site-specifically incorporate Cit into proteins.

Although Gln mutations have been used as surrogates for Cit^17^, Gln is smaller and does not accurately mimic the H-bonding patterns afforded by Cit. The in vitro translation systems or post-translational mutagenesis approaches that have been used to incorporate Cit are limited by their cumbersome nature, the need for specialized equipment, and for the latter approach, the need to incorporate a dehydroalanine at the site of modification, which is itself challenging and generates a mixture of d- and l-stereoisomers^18,19^. In addition, these strategies preclude the expression of site-specifically citrullinated proteins in living cells, and therefore, are ineffective for interpreting the downstream implications of this PTM. By contrast, Genetic Code Expansion (GCE) technologies enable the site-specific incorporation of unnatural amino acids (UAAs) into proteins using engineered aminoacyl-transfer RNA synthetase (aaRS)–tRNA pairs^20–24^. This technology has been used to genetically encode many important PTMs, enabling the expression of homogeneously modified protein at desired sites in living cells^25–28^. However, genetically encoding Cit using this technology has remained elusive so far.

In this work, we report the facile site-specific incorporation of Cit into proteins in mammalian cells using an *Escherichia coli*-derived engineered leucyl-tRNA synthetase (EcLeuRS)–tRNA_CUA_^EcLeu^ pair. This pair, in response to a nonsense codon (UAG), charges a photocaged-Cit, SM60 (Fig. 1b, c), into proteins expressed in HEK293T or EXPI293F cells. Subsequently, the photocage is removed with 365 nm ultraviolet (UV) to generate Cit in vitro or in living cells (Fig. 1c). To demonstrate proof of concept, we incorporate Cit at two well-known autocitrullination sites, R372 and R374, in PAD4 and elucidate how these modifications impact enzyme activity.

## Results

### Development of SM60: a photocaged-Cit

Envisioning that it may be challenging to develop an engineered aaRS that would selectively charge Cit, while discriminating against a nearly isostructural arginine, we hypothesized that these challenges could be overcome through a caging strategy. As such, we designed a photocaged-Cit (SM60, comprising an *o*-nitrobenzyl photocage on the Cit side chain), which is structurally distinct from the 20 canonical amino acids, but can be efficiently converted to Cit post-translationally (Fig. 1b, d). SM60 was synthesized over two steps from l-ornithine, and was characterized by ^1^H, ^13^C nuclear magnetic resonance (NMR) spectroscopy and mass spectrometry (MS) (Supplementary Figs. 1 and 2a). Using liquid chromatography-MS (LC-MS), we found that SM60 can be quantitatively converted to Cit in phosphate-buffered saline (PBS) supplemented with dithiothreitol (DTT) using 365 nm UV radiation for 5 min (Fig. 1d and Supplementary Fig. 3). The quantitative conversion was further supported by ^1^H NMR analysis, which shows the rapid disappearance of the benzylic protons of SM60 at 4.5 p.p.m. with increasing UV exposure and by the photodecaging of Fmoc-SM60 to Fmoc-Cit (Supplementary Figs. 4 and 5). To further investigate the feasibility of decaging SM60 on proteins, we synthesized an SM60-containing peptide, SM70 (Fig. 1b and Supplementary Fig. 2b), that contains residues 363–372 of PAD4 with SM60 at the 372 position, a known autocitrullination site (see below). Gratifyingly, SM70 also undergoes photodecaging to form the Cit-containing peptide (Supplementary Fig. 6), indicating that SM60 can be decaged in the presence of other amino acids.

### Genetically encoding SM60 in eukaryotes

**Fig. 2:**
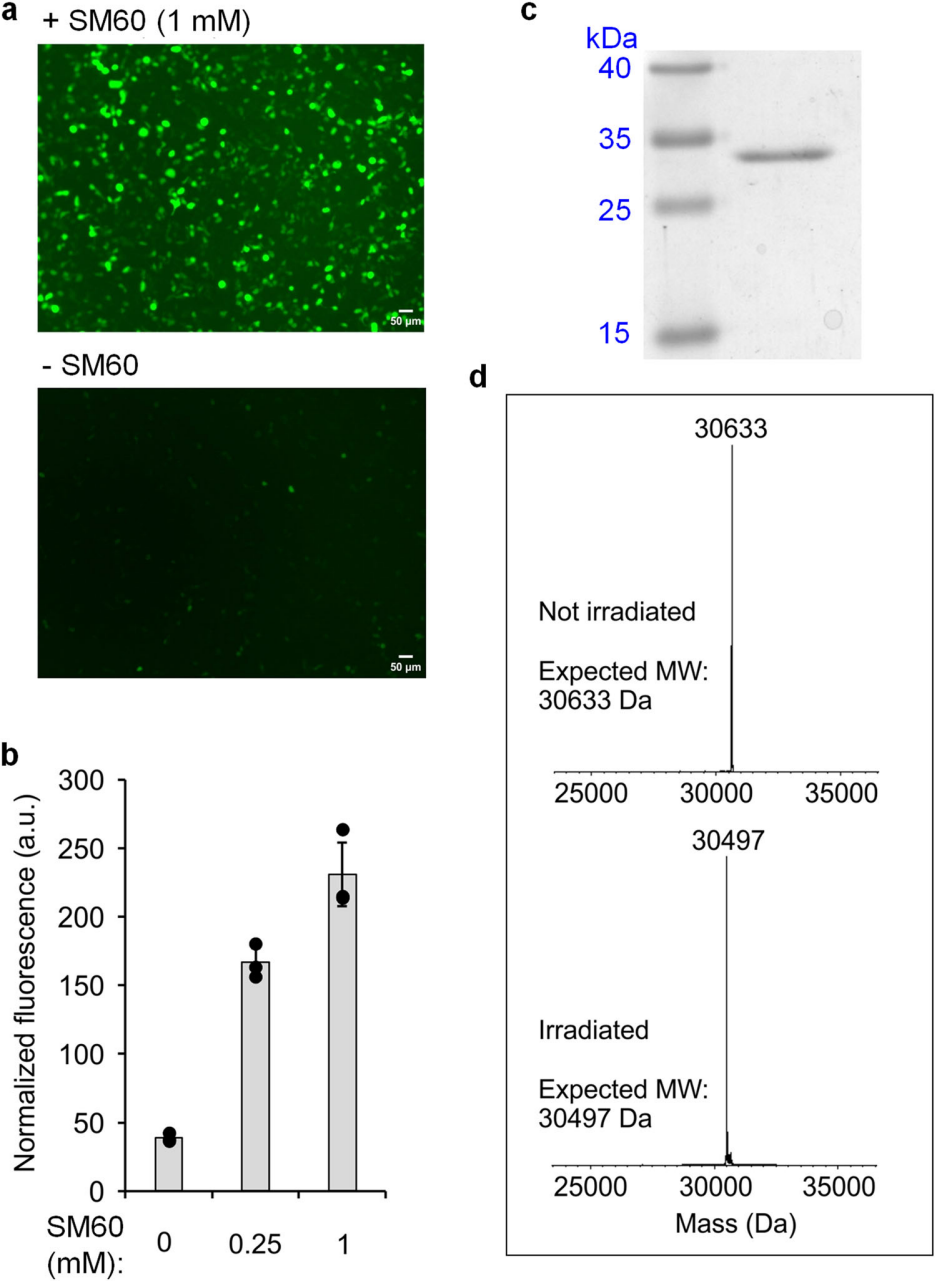
Site-specific incorporation of SM60 in EGFP and subsequent conversion to citrulline in HEK293T cells. **a** EGFP-39-TAG reporter expression by EcLeuRS-tRNA_CUA_^EcLeu^ pair in HEK293T cells in the presence of SM60 indicated by the fluorescence of EGFP. **b** Quantification of EGFP-39-TAG reporter expression efficiency in the presence of an increasing concentration of SM60. *n* = 3 independent experiments, data are presented as mean value ± SD. The solid circles represent data points for biologically independent replicates. Source data are provided as a Source Data file. **c** Coomassie stain of purified EGFP containing SM60. Full gel is given in Supplementary Fig. 7. **d** Deconvoluted mass spectrum of EGFP before and after 365 nm UV irradiation (1 min), indicating the presence of SM60 and citrulline, respectively, at position 39. Non-deconvoluted spectra are given in Supplementary Figs. 8 and 9.

Four different aaRS/tRNA pairs have been successfully engineered for incorporating UAAs in eukaryotic cells: bacteria-derived tyrosyl, tryptophanyl, and leucyl pairs and the archaea-derived pyrrolysyl pair^21–24,29,30^. The first two pairs are restricted to structural analogs of phenylalanine and tryptophan, respectively, precluding their use to genetically encode SM60. However, both the archaeal pyrrolysyl (PylRS/tRNA^Pyl^) and *E. coli* leucyl (EcLeuRS-tRNA_CUA_^EcLeu^) pairs have been engineered to charge UAAs structurally like SM60. Engineered aaRSs often exhibit substrate polyspecificity, that is, the ability to use several structurally analogous UAAs, while discriminating against the canonical amino acids. This property has provided a facile route to rapidly expand the repertoire of genetically encoded UAAs without having to engineer new aaRS mutants for each distinct substrate. To explore if such a polyspecific aaRS can accept SM60 as a substrate, we screened several existing PylRS and EcLeuRS mutants using an EGFP-39-TAG expression assay in HEK293T cells in the presence of their cognate amber suppressor tRNA. This screen identified an EcLeuRS mutant (M40I, Y499I, Y527A, and H529G in the active site, and T252A in the editing domain)^31^, which enabled the robust expression of the fluorescence reporter only when SM60 was supplemented in the medium (Fig. 2a, b). Purification of the resulting full-length EGFP using a C-terminal polyhistidine tag, followed by MS, showed a mass consistent with the successful incorporation of SM60 (Fig. 2c, d). Furthermore, UV irradiation of cells expressing EGFP-39-SM60 before lysis followed by protein purification and MS analysis afforded a single protein mass consistent with the complete deprotection and incorporation of Cit at position 39 of EGFP (Fig. 2d). Notably, SM60 is completely nontoxic (half-maximal effective concentration (EC_50_) > 10 mM) in HEK293T cells at the concentration used for nonsense suppression, that is, 1 mM (Supplementary Fig. 10A). Furthermore, a combination of SM60 and 365 nm UV exhibits an EC_50_ of 4.5 ± 0.2 mM for the inhibition of cell proliferation, indicating that the products of the photodecaging reaction (nitrosobenzaldehyde and Cit) have negligible cytotoxicity at the working concentration (Supplementary Fig. 10A).

### Site-specific incorporation of Cit in PAD4

**Fig. 3:**
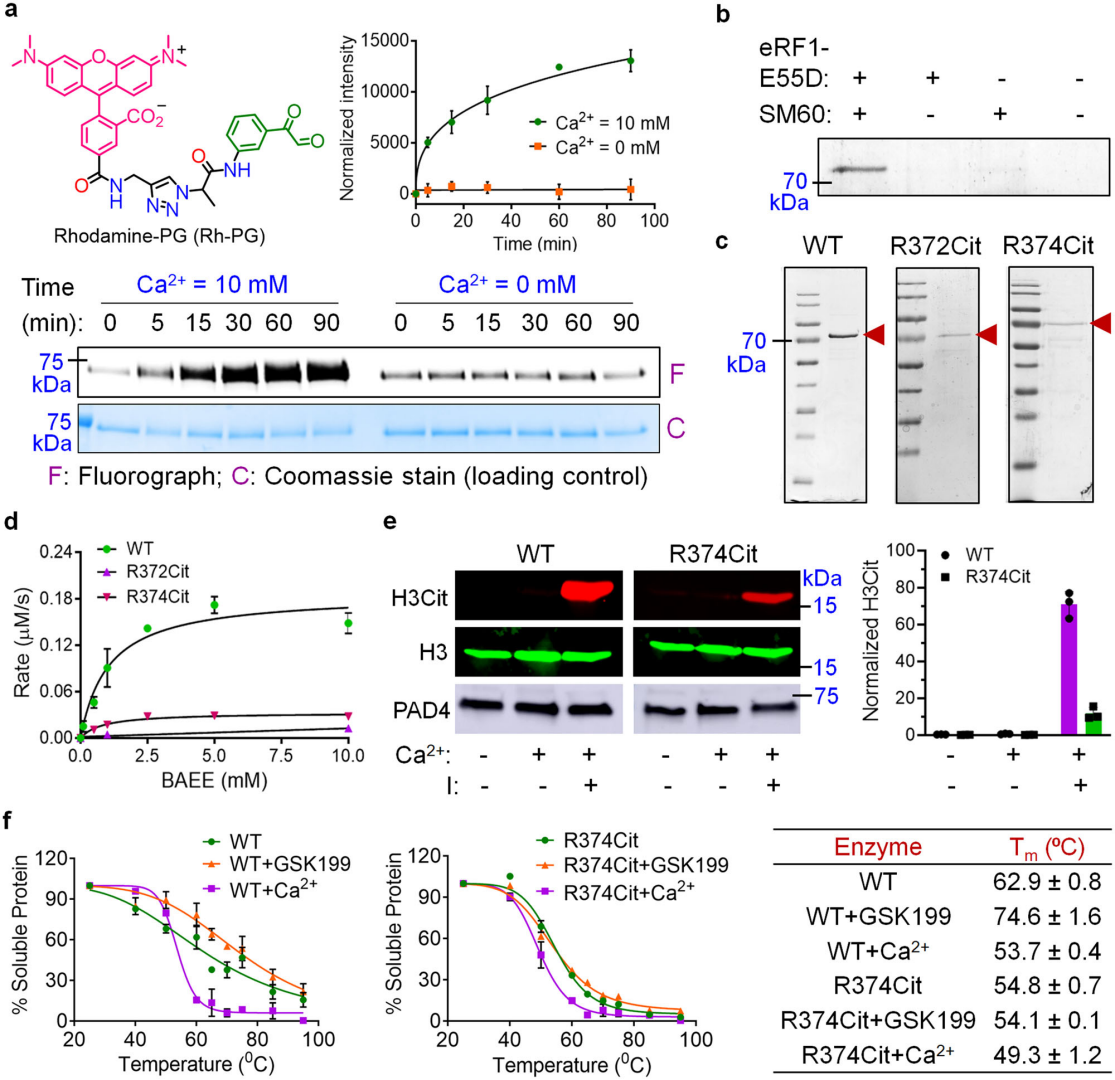
Autocitrullination of PAD4, incorporation of citrulline in PAD4 and implications thereof. **a** Chemical structure of Rh-PG and the fluorescence labeling of autocitrullinated PAD4. The bands at 0 min in the presence of calcium and at all the time points in the absence of calcium correspond to the basal levels of autocitrullination during the expression and purification of PAD4 from *E. coli*. *n* = 2 independent experiments, data are presented as mean value ± SD. **b** Expression of R372Cit PAD4 in the presence of engineered release factor, eRF1-E55D and SM60 in HEK293T cells, indicating the essential role of eRF1-E55D for efficient TAG suppression. **c** Coomassie stains for WT, R372Cit and R374Cit PAD4, indicating their purity. **d** Michaelis–Menten kinetics for WT, R372Cit, and R374Cit PAD4. *n* = 2 independent experiments, data are presented as mean value ± SD. **e** Western blot analysis of histone H3 citrullination in live HEK293T cells by WT and R374Cit PAD4. The normalization procedure is given in the “Methods” section. *n* = 3 independent experiments, data are presented as mean value ± SD. The samples derive from the same experiment and the blots were processed in parallel (see Supplementary Fig. 20c for a detailed explanation). **f** Thermal shift profiles of WT and R374Cit PAD4. Western blot images are given in Supplementary Fig. 17. The table indicates the melting temperatures (*T*_m_). *n* = 2 independent experiments, data are presented as mean value ± SD. The full gels corresponding to panels (**a**), (**b**), and (**e**) are given in Supplementary Fig. 20. Source data for panels (**a**) and (**d**–**f**) are provided as a Source Data file.

Having established our ability to site-specifically incorporate Cit into EGFP, we sought to exploit this technology to address the effect of autocitrullination on PAD4 activity. We focused on these studies because the effect of autocitrullination on PAD4 activity has been debated. While Andrade et al.^32^ reported that autocitrullination negatively impacts PAD4 activity, we showed that autocitrullination has little to no impact on PAD4 activity^17^. Using a Cit-specific fluorescent probe Rh-PG^13^, we confirmed that PAD4 autocitrullinates in the presence of Ca^2^^+^ in a time-dependent manner (Fig. 3a). We and others have previously mapped several autocitrullination sites in PAD4 (Supplementary Table 1 and Supplementary Fig. 11)^17,32^. While most of these sites are on the surface, the frequently observed R372 and R374 sites are present in the active site. Notably, the guanidinium groups on these two residues are only 3.5 Å from each other and the expected electrostatic repulsions are delicately balanced by H-bonding and salt-bridge interactions with D345. Moreover, R374 forms two H bonds with the small-molecule substrate, BAA^33^. Therefore, we expected that citrullination at these sites would significantly impact enzyme activity. To evaluate this hypothesis, we incorporated Cit at positions 372 and 374 in PAD4. Wild-type (WT) PAD4 and the 372 and 374 TAG mutants were separately cloned into a pAcBac3 plasmid^31,34^, which also encodes the mutant EcLeuRS and eight copies of the tRNA_CUA_^EcLeu^. Subsequently, these plasmids were transfected separately into HEK293T cells, and the PAD4 protein or its mutants (after irradiation to remove the photocage), were purified using a C-terminal polyhistidine tag. While WT PAD4 expression was robust (10 µg/10^7^ cells), yields for the mutants were very low, indicating poor suppression efficiency at these sites. The Chin group has recently reported a mutant eukaryotic release factor (eRF1-E55D) that can enhance TAG suppression efficiency in mammalian cells upon overexpression^35^. To explore if this strategy can overcome the low suppression efficiency at the target sites in PAD4, we cloned eRF1-E55D mutant under a cytomegalovirus promoter in the pIDTSMART vector. Indeed, co-transfection of this plasmid significantly improved the efficiency of nonsense suppression and enabled the purification of the desired mutants (2–4 µg/10^7^ cells, Fig. 3b, c). Since the +0.98 Da mass change upon citrullination is difficult to detect by intact MS analysis, we confirmed the incorporation of Cit at the desired position by LC-MS/MS analysis of the peptides resulting from Lys-C/Glu-C digestion of PAD4 (Supplementary Fig. 12, 13 and Supplementary Tables 2 and 3). Notably, we found that PAD4 expression does not cause any cytotoxicity in the HEK293T cells (Supplementary Fig. 10B). Using a similar procedure, we also expressed and purified WT PAD4 and the R374Cit mutant from EXPI293F cells that grow in suspension and are a highly scalable expression system (Supplementary Fig. 14).

### Activity of WT, R372Cit, R374Cit, and autocitrullinated PAD4

We first ensured that the biochemical activity and calcium dependence of WT PAD4 expressed from HEK293T cells (PAD4_Mam_) and *E. coli* (PAD4_Bac_) are similar (Supplementary Fig. 15a, b and Supplementary Table 4). Next, we determined kinetic values for the R372Cit and R374Cit mutants. Notably, both WT and the R374Cit mutant exhibited a time-dependent increase in Cit production (Supplementary Fig. 15c). By contrast, the R372Cit mutant produced only a negligible amount of Cit after 90 min. Furthermore, the rate of Cit production, indicated by the slope of the straight line, is significantly lower for the R374Cit mutant than WT PAD4 (Supplementary Fig. 15c). In agreement, the *k*_cat_/*K*_m_ values of the R374Cit and R372Cit mutants are 9- and 181-fold lower than that for WT PAD4 (Fig. 3d and Supplementary Table 4). These in vitro results led us to investigate the activity of WT and R374Cit PAD4 in live cells. Specifically, we overexpressed the two enzymes in HEK293T cells and evaluated their ability to citrullinate histone H3. Treatment of PAD4-overexpressing HEK293T cells with calcium and a calcium ionophore, that is, ionomycin, followed by western blot analysis indicated that WT PAD4 is six times more active than the R374Cit mutant for the citrullination of histone H3, consistent with the in vitro results (Fig. 3e).

Intrigued by these results, we sought to understand why the activity of these Cit-containing mutants is lower than WT PAD4. Kinetic studies indicate that the R374Cit mutant possesses a similar *K*_m_, but a 10-fold lower *k*_cat_ than WT PAD4 (Supplementary Table 4), suggesting a slow conversion of substrate to product. Furthermore, RFA, a PAD-targeted activity-based probe that covalently modifies the active site cysteine, C645^36^, fluorescently labeled only WT PAD4 when tested with both purified enzymes and enzyme-containing cell lysates (Supplementary Fig. 16). Based on these observations, we hypothesized that citrullination may induce local conformational changes within the active site, leading to very slow or no reaction between C645 and the guanidium group of the substrate, *N*^α^-benzoyl arginine ethyl ester (BAEE), or the fluoroacetamidine warhead on RFA. To investigate this possibility, we performed a thermal shift assay in the presence of a PAD4-selective ligand, GSK199, which binds to an allosteric pocket near the active site and H bonds to both D473 and H471^37^. Using this assay, we found the melting temperatures (*T*_m_) of WT and R374Cit PAD4 to be 62.9 and 54.6 °C, respectively (Fig. 3f and Supplementary Fig. 17). Despite having a lower T_m_, the R374Cit mutant is as stable as WT PAD4 at 37 °C (Supplementary Fig. 18). As expected, GSK199 increased the *T*_m_ of WT PAD4; however, it did not increase the *T*_m_ of the R374Cit mutant (Fig. 3f). These results indicate that GSK199 binds poorly to the R374Cit mutant, likely due to local conformational changes around the active site. However, the overall folding of the mutant is the same as WT since both the WT and R374Cit proteins exhibit a similar Δ*T*_m_ in the presence of calcium (Fig. 3f).

### Quantitative proteomics of PAD4 autocitrullination

As discussed earlier, conflicting reports indicated that autocitrullination can either inactivate the enzyme or have no effect. Since our present results suggest that autocitrullination should decrease PAD4 activity, we regenerated autocitrullinated PAD4 by incubating the enzyme in the presence of 10 mM CaCl_2_. Consistent with our previous observations, autocitrullinated PAD4 exhibits similar activity to control PAD4 (incubated in the absence of CaCl_2_) (Supplementary Fig. 15d). The activity loss for both autocitrullinated and control PAD4 in this experiment is likely due to the oxidation of C645 over time. Given that autocitrullination does not decrease enzyme activity, but citrullination of R372 and R374 does, we asked two questions. Are R372 and R374 the preferred sites of autocitrullination? Also, what fraction of PAD4 gets autocitrullinated? If only a small fraction of PAD4 is autocitrullinated, then this process should not impact the activity of uncitrullinated enzyme.

**Fig. 4:**
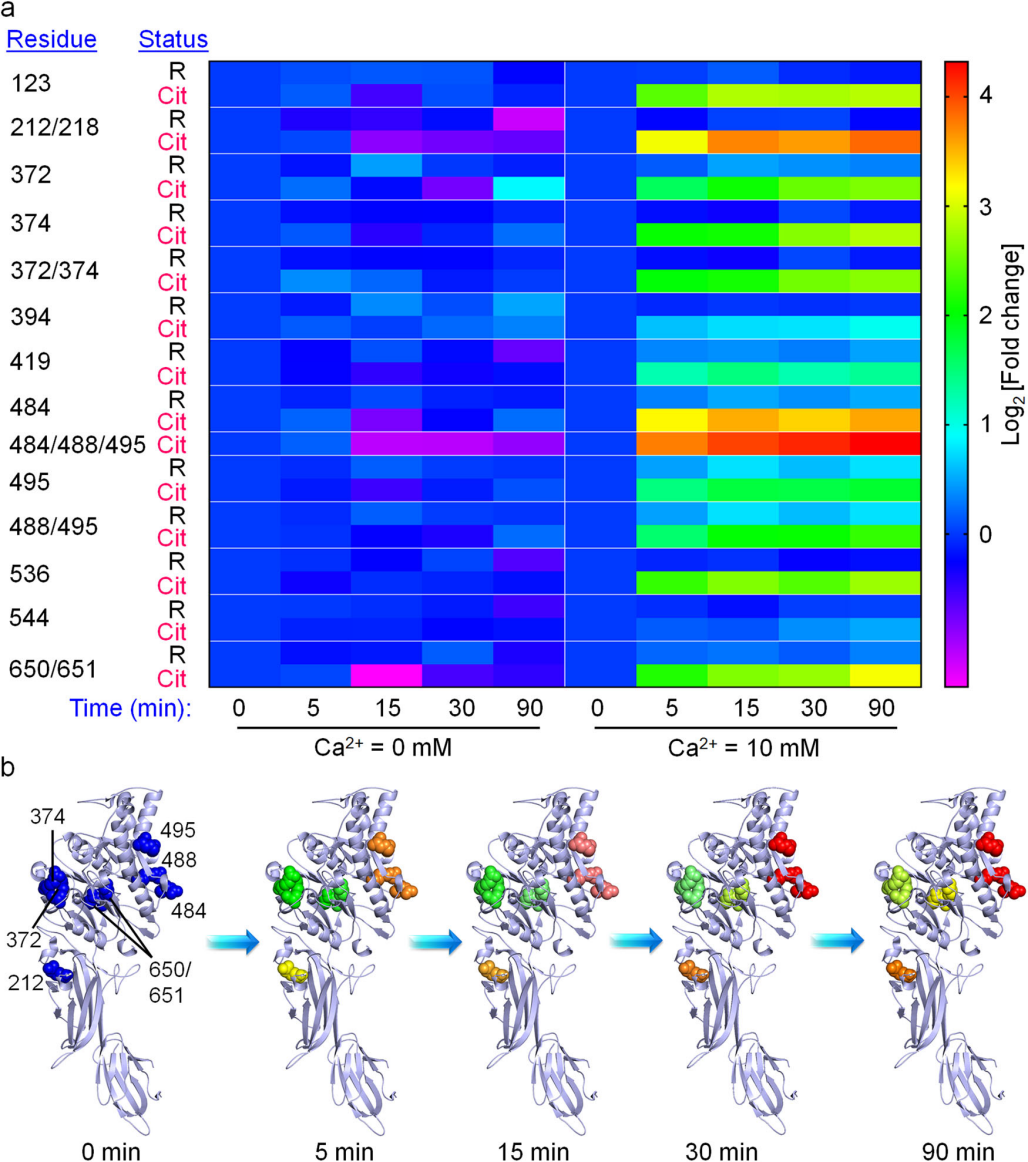
Autocitrullination sites in PAD4. **a** Heat map representing the time-dependent change in peptides containing arginine or citrulline at the indicated positions (autocitrullination sites). Ca^2+^-untreated samples are negative controls. **b** Time-dependent autocitrullination at the major sites. R218 site could not be shown because of disorder in that region (PDB: 1WDA)^33^. The increase in autocitrullination at these sites follows the same color code as in panel (**a**).

To answer these questions, we took a quantitative proteomics approach. PAD4 was autocitrullinated for various times and digested with Glu-C and Lys-C to maximize peptide coverage. The resulting peptides were then labeled with tandem mass tags (TMTs) and were subjected to tandem mass analysis (Supplementary Fig. 19). Enzyme incubated in the absence of calcium was used as the negative control. From this analysis, we identified 13 unique citrullination sites on PAD4 (Fig. 4a and Supplementary Tables 1 and 5). Notably, these exclude the previously reported R156, R205, R383, R609, and R639 and include two new sites, R650 and R651. Although both R372 and R374 residues show a time-dependent increase in citrullination, citrullination of arginines 212/218, 484/488/495, and 650/651 occurs at a much higher rate. For example, the extent of citrullination at arginines 212/218 and 484/488/495 is significantly higher at 5 min than that at the 372 and 374 sites after 90 min (Fig. 4a, b). These observations indicate that arginines 372 and 374 are not the preferred sites of autocitrullination. In addition, none of the observed autocitrullination sites exhibited a marked decrease in the arginine-containing parent peptide levels, which indicates that only a minor fraction of PAD4 undergoes autocitrullination, further explaining its nominal impact on the enzyme activity. Nonetheless, these results showcase how this technology provides the ability to systematically characterize the behavior of individual citrullinated isoforms of any protein—both in vitro and in living cells—providing a useful platform to understand the biology of this PTM.

## Discussion

Herein, we report the development of a genetic code expansion (GCE) technique for the site-specific incorporation of Cit into proteins in mammalian cells. Central to this technology is a photocaged-Cit (SM60) and an EcLeuRS/tRNA_CUA_^EcLeu^ pair that enables the incorporation of SM60 into proteins in response to a TAG nonsense codon with high fidelity and efficiency. Subsequently, the photocage is removed with 365 nm light to generate Cit. This technique overcomes several limitations of previous methods used to incorporate Cit, including in vitro translation, post-translational mutagenesis, and in vivo nonsense suppression by chemically acylated tRNAs^18,19,38^. For example, chemically acylated tRNAs are not readily synthesized, cannot be regenerated, and consequently give poor yields. By contrast, our approach provides a highly scalable expression platform that can be readily adapted by virtually any lab to site-specifically incorporate Cit into any mammalian protein, and thereby facilitate cellular studies to understand the downstream implications of this PTM.

Specifically, we incorporated Cit at two known autocitrullination sites, R372 and R374, in PAD4. Kinetic studies indicate that the R374Cit and R372Cit mutants are 9- and 181-fold less active than WT PAD4. Detailed studies indicate that citrullination induces local conformational changes within the active site that leads to a slow reaction between C645 and the guanidium group of the substrate, the first step in the catalytic cycle. While these results indicate that citrullination of R372 and R374 would decrease PAD4 activity, we found that autocitrullination does not impact the enzymatic activity. Quantitative proteomics studies indicate that 212/218 and 484/488/495, and not 372 and 374, are the preferred sites of citrullination. While faster autocitrullination of arginines 212/218 and 484/488/495 is likely due to their residence at the surface of PAD4, upon citrullination, they may expose deeply buried autocitrullination sites by conformational changes. Efforts are currently underway to elucidate the effect of citrullination at these major sites, particularly the 484/488/495 residues because they are present at the interface of the head-to-tail PAD4 dimer that is known to alter enzymatic activity.

Since it is well established that citrullination is critical for many physiological processes, as well as in disease pathology, our method will provide a direct and accessible approach to understand the biology of this PTM at the molecular level. For example, histone H3 citrullination at R26 leads to the transcriptional activation of >200 genes in estrogen receptor-positive breast cancer cells and inhibits the methylation of the neighboring K27 residue by 30,000-fold^15^. However, the mechanism of negative crosstalk between these two PTMs remains poorly understood. In addition, we recently showed that SERPINs, nicotinamide *N*-methyl transferase (NNMT), and pyruvate kinase isoform M2 (PKM2) are citrullinated in patients suffering from RA. Notably, the citrullination of the SERPINs and NNMT dramatically abolishes their enzymatic activity, while citrullinated PKM2 exhibits 2–3-fold higher activity than the WT enzyme^14^. However, the underlying reasons behind such biochemical phenomenon are unclear. Finally, citrullination has been reported to impact NET formation, pluripotency, and efficient elongation by RNA PolII, but, again, the underlying mechanisms remain unclear^1,8^. With this GCE technology, it is possible to incorporate Cit on demand and mechanistically address how this PTM impacts these fundamental biological processes and pathways.

## Methods

### Materials and general methods

*N*^α^-Fmoc-*N*^δ^-l-ornithine hydrochloride, 2-(1*H*-benzotriazol-1-yl)-1,1,3,3-tetramethyluronium hexafluorophosphate (HBTU), 1-hydroxybenzotriazole (HOBt), and other Fmoc-protected amino acids were purchased from Chem-Impex International Inc. 1-(Isocyanatomenthyl)-2-nitrobenzene (1 M solution in toluene) was purchased from Ellanova Laboratories. Triethylamine, trifluoroacetic acid, anhydrous dichloromethane, anhydrous dimethylformamide, piperidine, and high-performance liquid chromatography (HPLC)-grade acetonitrile were bought from Sigma-Aldrich. Halt protease inhibitor cocktail (EDTA-free), Universal nuclease, Ni-NTA resin, Pierce™ Peptide Desalting Spin Columns (catalog no. 89852), Pierce™ Quantitative Fluorometric Peptide Assay kit (catalog No. 23290), and TMT10plex™ Isobaric Label Reagent (catalog no. 90110) were obtained from Thermo Fisher Scientific. Deuterated solvents were purchased from Cambridge Isotope Laboratories. Plasmid purification kit was bought from Bio Basic Canada Inc. The TOP10 *E. coli* strain was used for plasmid construction and propagation. The cells were grown in LB liquid medium with 100 µg/mL ampicillin or 50 µg/mL kanamycin. Primers were synthesized by Integrated DNA Technologies (Coralville, IA). Restriction enzymes (NEB, Beverly, MA), Phusion Hot Start II DNA polymerase (Fisher Scientific, MA), and T4 DNA ligase (Enzymatics, Beverly, MA) were used for plasmid construction following the manufacturer’s protocols. White light and fluorescence imaging of HEK293T cells expressing the EGFP-39-TAG reporter was performed using a Zeiss AX10 microscope. Rabbit polyclonal anti-PAD4 (catalog no. ab50332), rabbit polyclonal anti-histone H3 (Cit R2,8,17) (catalog no. ab5103), and mouse monoclonal anti-histone H3 (catalog no. ab10799) were obtained from Abcam and all these antibodies were used at 1:1000 dilution. EXPI293F cells, EXPI293^TM^ expression medium, and ExpiFectamine^TM^ 293 Transfection Kit were obtained from Gibco. Dulbecco’s modified Eagle’s medium (DMEM), fetal bovine serum, and antibiotic–antimycotic (100×) solution were obtained from Gibco and were used for HEK293T (source: ATCC) cell maintenance. Mass spec grade Lys-C (catalog no. VA117A) and sequencing grade Glu-C (catalog no. V165A) were obtained from Promega. ^1^H and ^13^C NMR spectra were recorded in dimethyl sulfoxide-*d*_6_ (DMSO-*d*_6_) as solvent using a Bruker 500 MHz NMR spectrometer. Chemical shift values are cited with respect to SiMe_4_ (TMS) as the internal standard. All the compounds were purified by reverse-phase HPLC using a semi-preparative C18 column (Agilent, 21.2 mm × 250 mm, 10 µm) and a water/acetonitrile gradient supplemented with 0.05% trifluoroacetic acid. Fluorographs were recorded using a Typhoon scanner with excitation/emission maxima of ∼546/579, respectively. WT PAD4 obtained from a bacterial expression system (PAD4_Bac_) was expressed and purified as reported earlier^39^.

### Synthesis of SM60

SM60 (Fig. 1b) was synthesized as depicted in Supplementary Fig. 21. Briefly, Fmoc-Orn-OH (1 g, 2.6 mmol) was suspended in anhydrous dichloromethane and triethylamine (0.7 mL, 5.2 mmol) was added to it. 1-(Isocyanatomenthyl)-2-nitrobenzene (1 M solution in toluene) (2.6 mL, 2.6 mmol) was then added dropwise and the reaction mixture was stirred at room temperature for 12 h. Excess triethylamine and dichloromethane was then evaporated under reduced pressure to afford a yellowish-brown semisolid that was used for the subsequent step without further purification. The crude product was dissolved in 1:4 piperidine/dimethylformamide (10 mL) and was stirred at room temperature for 30 min. The reaction mixture was then vigorously stirred with excess hexane and the hexane layer was decanted off. Washing with hexane was repeated several times to remove most of the dimethylformamide. The pale yellow semisolid obtained thereafter was purified by reverse-phase HPLC using a pre-packed C18 column and a water/acetonitrile (supplemented with 0.05% trifluoroacetic acid) gradient as eluent to afford SM60 as a white solid (overall yield: 60%). SM60 was thoroughly characterized with ^1^H and ^13^C NMR spectroscopy and MS. ^1^H NMR (500 MHz, DMSO-*d*_*6*_): δ 8.18 (s, 3H), 7.94 (dd, *J* = 9 Hz, 1H), 7.64–7.67 (m, 1H), 7.43–7.48 (m, 2H), 6.48 (t, *J* = 5 Hz, 1H), 6.24 (t, *J* = 5 Hz, 1H), 4.4 (d, *J* = 10 Hz, 2H), 3.85 (s, 1H), 2.93–2.97 (m, 2H), 1.61–1.75 (m, 2H), 1.40–1.49 (m, 1H), 1.31–1.39 (m, 1H); ^13^C NMR (500 MHz, DMSO-*d*_*6*_): δ 171.5, 158.5, 148.3, 136.6, 134.2, 130.0, 128.4, 124.9, 52.3, 40.7, 39.1, 28.0, 26.2; HRMS (*m*/*z*): [M + H]^+^ calcd. for C_13_H_18_N_4_O_5_: 311.1355; found, 311.1337.

### Synthesis of SM70

SM70 (Fig. 1b) was synthesized using an automated solid-phase peptide synthesizer (PS3, Protein Technologies, Inc.) by following the manufacturer’s protocol. Briefly, Fmoc-Lys(Boc)-Wang resin (350 mg, 0.2 mmol) was taken in a 30 mL glass reaction vessel, and Fmoc-SM60 (425 mg, 0.8 mmol), Fmoc-Ala-OH (249 mg, 0.8 mmol), Fmoc-Ser(tBu)-OH (306 mg, 0.8 mmol), Fmoc-Asp(OtBu)-OH (329 mg, 0.8 mmol), Fmoc-Phe-OH (310 mg, 0.8 mmol), Fmoc-Val-OH (272 mg, 0.8 mmol), Fmoc-Val-OH (272 mg, 0.8 mmol), Fmoc-Trp(Boc)-OH (421 mg, 0.8 mmol), Fmoc-Leu-OH (283 mg, 0.8 mmol), and Fmoc-Thr-OH (318 mg, 0.8 mmol) were taken in separate amino acid vials. HBTU (303 mg, 0.8 mmol) and HOBt (108 mg, 0.8 mmol) were then added to each amino acid vial. *N*-methylmorpholine (0.4 M in *N*,*N*-dimethylformamide (DMF)) was used as a base for the activation of the carboxylic acid group with HBTU and HOBt. The N-terminal Fmoc protecting group on each amino acid was removed with 20% piperidine in DMF. Each peptide coupling reaction was carried out for 1 h. Once all the amino acids were coupled, the resin was transferred to a synthetic column, thoroughly washed with DMF and dichloromethane (DCM), and the peptide was cleaved from the resin by treating with a cleavage cocktail (95% trifluoroacetic acid, 4.5% triisopropylsilane, 0.5% water, 10 mL) at room temperature for 30 min with constant mixing. The flow-through was collected and the resin was washed 2–3 times with trifluoroacetic acid. The combined flow-through and trifluoroacetic acid washes were then treated with 8–10 times excess cold diethyl ether to precipitate the peptide. Excess ether and trifluoroacetic acid was slowly evaporated by purging nitrogen. The crude peptide was purified by reverse-phase HPLC using a pre-packed C18 column and a water/acetonitrile (supplemented with 0.05% trifluoroacetic acid) gradient to afford SM70 as a white solid (overall yield: 15%). SM70 was characterized by electrospray ionization (ESI) MS, HRMS (*m*/*z*) calculated for C_69_H_100_N_16_O_19_ [M + 2H]^2+^: 729.3753, found 729.3730.

### Construction of plasmids

The previously reported pAcBac3-EcLeuTAG-EGFP-39-TAG plasmid was used to construct additional plasmids^31^. The EGFP-39-TAG reporter in this plasmid was replaced with WT PAD4 gene using flanking SfiI restriction site to create pAcBac3-EcLeuPLRS1TAG-PAD4WT. For incorporation of *o*-nitrobenzyl-Cit, TAG codons were introduced at desired sites by site-directed mutagenesis using overlap-extension polymerase chain reaction. For the R372TAG mutation, PAD4WT-forward + PAD4 R372TAG inner reverse primer and PAD4 R372TAG inner forward primer + PAD4WT-Reverse was used to create mutated 5ʹ and 3ʹ fragments of PAD4 (for primer sequences see Supplementary Table 6). The full-length PAD4 R372TAG insert was generated by the overlap extension of these fragments. The pAcBac3-EcLeuPLRS1TAG-PAD4 R374TAG was constructed similarly using the PAD4 R374TAG inner forward primer and PAD4 R374TAG inner reverse primer. The open-reading frame for eRF1-E55D^35^ was purchased from Integrated DNA Technologies as a gBlock. The gBlock was amplified with eRF1-forward and eRF1-reverse primers (Supplementary Table 6) and was introduced into pIDTSMART vector with flanking *Nhe*I and *Xho*I restriction sites to create pIDTSMART eRF1-E55D.

### EGFP and PAD4 expression from HEK293T cells

HEK293T cells were maintained at 37 °C in a humidified incubator supplemented with 5% CO_2_. Cells were seeded at 9 × 10^6^ cells per 10 cm plate 24 h before transfection. EGFP and WT PAD4 transfections were performed by incubating 10 µg plasmid DNA, 50 µL polyethylenimine (PEI) (1 mg/mL; Polysciences, Warrington, PA), and 180 µL DMEM for 10 min at room temperature, followed by adding the solution dropwise to the culture medium of the cells. For EGFP-39-TAG expression, 1 mM SM60 was added to the culture medium following transfection. For SM60 incorporation into PAD4 at positions 372 and 374, 12 µg of PAD4 R372TAG or PAD4 R374TAG and 8 µg of pIDTSMART eRF1-E55D plasmids were incubated with a mixture of 100 µL PEI and 180 µL DMEM for 10 min at room temperature before adding to cells. SM60 was added at the same time to a final concentration of 1 mM, and 2 mM sodium butyrate was added to enhance protein expression. The full gels for the EGFP and PAD4 expression are given in Supplementary Figs. 7 and 20b.

### EGFP fluorescence analysis

EGFP fluorescence was analyzed 48 h after transfection. DMEM was exchanged with PBS and the plates were irradiated at 365 nm (120 W, 10 cm × 10 cm LED array; Larson Electronics) for 75 s at 4 °C to decage SM60. Cells were then harvested and resuspended in 600 µL CelLytic M buffer (Sigma, St. Louis, MO) with Halt Protease inhibitor Cocktail (Thermo Scientific, Waltham, MA) and Pierce Universal Nuclease for Cell Lysis (Fisher Scientific, Hampton, NH). Lysates were clarified by centrifugation at 16,000 × *g* for 10 min and 100 µL of supernatant was transferred to a clear-bottom 96-well plate for fluorescence measurement. Fluorescence was measured using a Zeiss AX10 microscope (Ex. 488 nm; Em. 534 nm). All the experiments were performed at least in duplicate.

### LC-MS analysis of EGFP

LC/MS analyses of the purified EGFP were performed on Agilent Technologies 6230 TOF LC/MS with Aerns 3.6 µm WIDEEPORE XB-C8 20 LC column 100 × 4.6 mm^2^.

### Cytotoxicity of SM60 in HEK293T cells

HEK293T cells were seeded (2 × 10^4^ cells/well) on a 96-well plate and grown in DMEM (supplemented with 10% heat-inactivated fetal bovine serum, 100 U/mL penicillin, 100 µg/mL streptomycin) for 48 h. Cells were then treated with DMSO (for control) or various concentrations of SM60. In a separate experiment, cells were briefly (3 min) irradiated with 365 nm UV following SM60 treatment and were allowed to grow at 37 °C for 48 h. Cell viability was measured using CellTiter-Blue® (Promega) by following the manufacturer’s protocol. Equation (1),

1 $$Y = {\mathrm{Bottom}} + \left( {{\mathrm{Top}} - {\mathrm{Bottom}}} \right)/[1 + 10^{\left( {\left( {\log {{\mathrm{EC}}_{50} }- X} \right)\times {\mathrm{Hillslope}}} \right)}],$$

was used to fit a ten-point dose–response curve to determine the EC_50_ values for inhibition of cell proliferation using GraphPad Prism 8.0. Top and Bottom are plateaus of the dose–response curve, *X* is the log of SM60 concentration, Hillslope is the slope factor or Hill slope. All the experiments were performed in triplicate.

### Overexpression of WT and mutant (R374Cit) PAD4 in EXPI293F cells

EXPI293F cells were co-transfected with the engineered *LeuRS/tRNA*^*Leu*^*/PAD4* and *eRF1* genes using ExpiFectamine^TM^ 293 transfection kit by following the manufacturer’s protocol. Briefly, EXPI293F cells were grown to 2.9 × 10^6^ density in the EXPI293^TM^ expression medium (9 mL) at 37 °C under 5% CO_2_ atmosphere. pAcBac3 plasmid (6 µg) encoding the genetically engineered LeuRS, tRNA^Leu^, and WT PAD4 or mutant PAD4 (containing TAG mutation at the 374 position) and the plasmid encoding release factor, pIDTSMART eRF1-E55D (4 µg), were resuspended in 0.5 mL opti-MEM^TM^ I reduced serum media. ExpiFectamine^TM^ (27 µL) was also resuspended in 0.5 mL opti-MEM^TM^ media and then the plasmid mixture was slowly added to ExpiFectamine^TM^ solution. This mixture was incubated at room temperature for 20 min to form the DNA polyplex. Then the DNA polyplex (1 mL) was slowly transferred to the EXPI293F cell suspension (9 mL) and SM60 (100 µL of 100 mM stock in DMSO, 1 mM final) was added to the medium. The cells were then incubated at 37 °C under 5% CO_2_ atmosphere for 24 h with constant shaking. Transfection enhancers 1 (50 µL) and 2 (500 µL) (supplied with the ExpiFectamine^TM^ 293 transfection kit) were then added to the cell suspension and the cells were further grown at 37 °C under 5% CO_2_ atmosphere for 48 h with constant shaking. Cells were then harvested, washed with cold Dulbecco’s phosphate-buffered saline (DPBS), resuspended in 5 mL DPBS, and were taken in a cell culture dish (100 mm × 20 mm, Corning). Cells were then irradiated with 365 nm light for 5 min using a photoreactor (Luzchem) containing 14 UV-A lamps (8 W each). After UV-A irradiation, cells were harvested, resuspended in 1 mL in DPBS (containing 1× Halt protease inhibitor cocktail), and lysed using a probe sonicator. Overexpression of WT and mutant PAD4 was confirmed by western blot analysis of the EXPI293F cell lysate using rabbit polyclonal anti-PAD4 antibody.

### Purification of WT and mutant PAD4 from HEK293T cells

Cells from a 10 cm plate were harvested 48 h after transfection. For cells that overexpressed proteins containing SM60, media were exchanged with PBS and the plates were irradiated at 365 nm (120 W, 10 cm × 10 cm LED array (Larson Electronics)) for 75 s at 4 °C to decage SM60 right before harvesting. The cells were resuspended in 600 µL CelLytic M buffer (Sigma, St. Louis, MO) with Halt protease inhibitor cocktail (Thermo Scientific, Waltham, MA) and Pierce universal nuclease for cell lysis (Fisher Scientific, Hampton, NH). After a 10-min incubation at room temperature, 1.2 mL of equilibration buffer (20 mM Na_2_HPO_4_, 300 mM NaCl, 10 mM imidazole pH 7.4) was added. Lysate was clarified by centrifugation at 16,000 × *g* for 10 min at 4 °C. The clarified cell-free extract was subjected to Ni-NTA affinity chromatography using HisPur resin (Fisher Scientific, Hampton, NH) following the manufacturer’s protocol.

### Purification of WT and mutant PAD4 from EXPI293F cells

Following UV-A irradiation, cells were resuspended in lysis buffer (20 mM Tris-HCl pH 8.0, 400 mM NaCl, 10 mM imidazole, 2 mM DTT, 10% glycerol, 1× protease inhibitor cocktail, 1× universal nuclease) and lysed using a probe sonicator. The lysate was centrifuged at 18,000 × *g* for 20 min at 4 °C and the supernatant was incubated with Ni-NTA agarose beads (prewashed with the lysis buffer) for 30 min at 4 °C on an end-over-end shaker. The beads were then transferred to a synthetic column and washed sequentially with buffer 1 (20 mM Tris-HCl pH 8.0, 400 mM NaCl, 50 mM imidazole, 2 mM DTT, 10% glycerol) and buffer 2 (20 mM Tris-HCl, pH 8.0, 400 mM NaCl, 75 mM imidazole, 2 mM DTT, 10% glycerol). Finally, PAD4 was eluted from the beads using elution buffer (20 mM Tris-HCl pH 8.0, 500 mM NaCl, 300 mM imidazole, 2 mM DTT, 10% glycerol). Eluted PAD4 was then dialyzed against 20 mM Tris-HCl, pH 8.0, 500 mM NaCl, 2 mM DTT, 10% glycerol using a dialysis cassette with a molecular weight cutoff of 3.5 kDa.

### Digestion of the PAD4R372Cit and PAD4R374Cit mutants, and LC-MS/MS analysis

The R372Cit and R374Cit mutants (15 µg) were independently resuspended in 20 mM Tris-HCl (200 µL, pH 7.4) and then trichloroacetic acid (20% final) added to the samples. The resultant cloudy mixture was vortexed vigorously and was kept at −20 °C for 30 min to precipitate the protein. Then, the mixture was centrifuged at 21,000 × *g* for 30 min at 4 °C. The pellet was washed with cold acetone, resuspended in 30 µL of 8 M urea in PBS (pH 7.4) with sonication, and 70 µL of 100 mM ammonium bicarbonate was added to the solution. Then, 1.5 µL of 1 M DTT was added and the solution was incubated at 65 °C for 20 min. A measure of 2.5 µL of freshly prepared 500 mM iodoacetamide was added and the solution was incubated at room temperature for 30 min in dark. The alkylation reaction was diluted by the addition of 120 µL PBS (pH 7.4). Lys-C (1 µL of a 1 µg/µL solution, reconstituted in water) and Glu-C (2 µL of a 0.5 µg/µL solution, reconstituted in water) was then added to the samples and the mixture was incubated at 37 °C for 16 h. The proteolysis reaction was terminated by adding 10 µL of formic acid. The peptide mixture was then desalted using Pierce^TM^ desalting C18 spin columns following the manufacturer’s protocol and the samples were analyzed by LC-MS/MS as described below.

### Cytotoxicity of PAD4 expression in HEK293T cells

HEK293T cells were seeded (2 × 10^4^ cells/well) on 96-well plate and were allowed to grow in DMEM (supplemented with 10% heat-inactivated fetal bovine serum, 100 units/mL penicillin, 100 µg/mL streptomycin) for 48 h. Cells were then transfected with appropriate plasmids (120 ng PAD4 plasmid and 80 ng pIDTSMART eRF1-E55D for each well) using Lipofectamine 2000 (500 ng for each well) in Opti-MEM medium (Gibco). After 5 h, the medium was changed to DMEM and DMSO (for WT PAD4) or SM60 (0.25, 5, and 1 mM for R374Cit PAD4) was added. Cells were then allowed to grow at 37 °C for 48 h. Cell viability was measured before and after photodecaging (by UV treatment for 3 min) using CellTiter-Blue® (Promega) by following the manufacturer’s protocol. Untransfected cells served as the control. All the experiments were performed in triplicate.

### Time-dependent Cit production

PAD4 (6 µL of a 1 µM stock, 100 nM final) was added to a pre-warmed (10 min, 37 °C) reaction mixture (60 µL final) containing BAEE (10 mM), CaCl_2_ (10 mM), Tris-HCl (100 mM, pH 7.4), NaCl (50 mM), and DTT (2 mM). This mixture was incubated at 37 °C for 0, 30, 50, and 90 min, after which the reaction was stopped by flash freezing with liquid nitrogen. The production of Cit at various time points was quantitated by the standard COLDER assay^40,41^. The time-dependent production of Cit by PAD4 was fit to the equation for a straight line using GraphPad Prism 8.0. All the reactions were performed at least in duplicate.

### Michaelis–Menten kinetics of WT and mutant PAD4

PAD4 (50 nM final for WT, 75 nM final for R372Cit, or 100 nM final for R374Cit) was added to a pre-warmed (10 min, 37 °C) reaction mixture (60 µL final) containing various concentrations of BAEE (0, 0.5, 1, 2.5, 5, and 10 mM), CaCl_2_ (10 mM), Tris-HCl (100 mM, pH 7.4), NaCl (50 mM), and DTT (2 mM). This mixture was incubated at 37 °C for 45 min (for WT), 90 min (for R374Cit), and 120 min (for R372Cit), followed by quenching with liquid nitrogen. The rate of Cit formation at various BAEE concentrations was quantified with the COLDER assay^40,41^. The rates were plotted against the BAEE concentration and were fit to the Michaelis–Menten equation using GraphPad Prism 8.0. All the reactions were performed at least in duplicate.

### Calcium dependence of WT PAD4

PAD4_Bac_ (purified from bacterial expression system) and PAD4_Mam_ (purified from mammalian expression system) (6 µL of 0.5 µM stock, 50 nM final) were added to separate pre-warmed (10 min, 37 °C) reaction mixtures (60 µL final) containing BAEE (10 mM), various concentrations of CaCl_2_ (0, 0.25, 0.5, 1, 2.5, 5, and 10 mM), Tris-HCl (100 mM, pH 7.4), NaCl (50 mM, and DTT (2 mM). This mixture was incubated at 37 °C for 45 min, followed by flash freezing with liquid nitrogen. The production of Cit at various concentrations of CaCl_2_ by PAD4 was quantified with the COLDER assay^40,41^. The calcium dependence of Cit production by PAD4 was fit to Eq. (2),

2 $$v = {V}_{\max }{\,}^\ast \!\left[ {{\mathrm{Ca}}^{2 + }} \right]^{h}{\mathrm{/}}\left( {K_{0.5}^{h}{\mathrm{ + }}\left[ {{\mathrm{Ca}}^{2 + }} \right]^{h}} \right)$$

using GraphPad Prism 8.0, where *v* is the velocity of the reaction, *V*_max_ is the maximum velocity of the reaction, [Ca^2+^] is the concentration of calcium, *h* is the hill slope, and *K*_0.5_ is the calcium concentration that gives half-maximal velocity. All the reactions were performed at least in duplicate.

### Effect of autocitrullination of WT PAD4 on the enzymatic activity

PAD4_Bac_ (0.5 µM final) was added to a pre-warmed (10 min, 37 °C) solution containing CaCl_2_ (0 or 10 mM), Tris-HCl (100 mM, pH 7.4), NaCl (500 mM), and DTT (2 mM). At various time points (0, 5, 15, 30, 60, and 90 min), 6 µL of this reaction mixture was removed and was added to a pre-warmed (10 min, 37 °C) reaction mixture (10 mM BAEE, 10 mM CaCl_2_, 100 mM Tris, pH 7.4, 500 mM NaCl, 2 mM DTT, with a final volume of 60 µL). After 45 min, the reaction mixture was flash frozen with liquid nitrogen. The production of Cit at various time points was quantified with the COLDER assay^40,41^. The loss in activity of PAD4 over time was fit into single exponential decay using GraphPad Prism 8.0. All the reactions were performed at least in duplicate.

### Rh-PG labeling of autocitrullinated PAD4

Rh-PG labeling of autocitrullinated PAD4 was performed as reported earlier with minor modifications^13^. PAD4 (0.5 µM final) was added to a pre-warmed (10 min, 37 °C) reaction mixture containing CaCl_2_ (0 or 10 mM), Tris-HCl (100 mM, pH 7.4), NaCl (500 mM), and DTT (2 mM). At various time points (0, 5, 15, 30, 60, and 90 min), the reaction was stopped by flash freezing in liquid nitrogen. The samples were thawed, and trichloroacetic acid (20% final) and Rh-PG (80 µM final) were sequentially added to it. The reaction mixture was incubated at 37 °C for 1 h. Then, the reaction was quenched with Cit (100 mM final) dissolved in 50 mM Tris-HCl (pH 7.4) and the mixture was further incubated at 37 °C for 30 min. The samples were placed at −20 °C for 30 min and the precipitated proteins were collected by centrifugation (21,000 × *g* for 30 min) at 4  °C. The protein pellet was washed with cold acetone and dried. Then, the pellet was dissolved in 40 µL buffer containing 100 mM arginine, 20 mM Tris-HCl (pH 7.4), 1% sodium dodecyl sulfate (SDS), and 10 µL 5× SDS loading dye. The proteins were separated by SDS-polyacrylamide gel electrophoresis (SDS-PAGE) and the fluorescently labeled bands were visualized by scanning the gel in a typhoon scanner (excitation and emission maxima ~546 and 579 nm, respectively). The fluorescent intensities of protein bands were quantified using ImageJ 1.52v and were normalized against the coomassie intensities that indicate the amount of protein present in each lane. All the reactions were performed at least in duplicate. The full gel for the Rh-PG labeling of autocitrullinated PAD4 is given in Supplementary Fig. 20a.

### RFA labeling of WT and R374Cit PAD4

RFA labeling of PAD4 was carried out by following a protocol similar to that established for PAD1 and PAD2^10,11^. Briefly, PAD4 (100 nM final) was added to pre-warmed (10 min, 37 °C) reaction mixture (100 mM Tris pH 7.4, 500 mM NaCl, 0 or 10 mM CaCl_2_, and 2 mM DTT in a final volume of 30 µL) containing RFA (200 nM final). After incubating at 37 °C for 2 h, the reaction mixture was quenched with 5× SDS-PAGE loading dye and boiled at 95 °C for 10 min. The proteins were separated by SDS-PAGE using a 4–20% gradient gel and fluorescently labeled proteins were visualized by scanning the gel in a typhoon scanner (excitation and emission maxima ~546 and 579 nm, respectively). The fluorescent intensities of protein bands were quantified using ImageJ 1.52v. All the reactions were performed at least in duplicate.

### RFA labeling of WT and R374Cit PAD4 in EXPI293F lysate

RFA (10 µM) was added to a pre-warmed (10 min, 37 °C) reaction mixture (2 mg/mL EXPI293F lysate containing WT or R374Cit or R372Cit PAD4 in 1× PBS, 2 mM CaCl_2_, and 2 mM DTT in a final volume of 50 µL) and the mixture was incubated at 37 °C for 2 h. The reaction was quenched with 5× SDS-PAGE loading dye and was boiled at 95 °C for 10 min. The proteins were separated on a 4–20% SDS-PAGE gel and the fluorescently labeled proteins were visualized by scanning the gel in a typhoon scanner (excitation and emission maxima ~546 and 579 nm, respectively). The fluorescent intensities of protein bands were quantified using ImageJ 1.52v. All the reactions were performed at least in duplicate.

### Histone H3 citrullination in live HEK293T cells

HEK293T cells were seeded (4 × 10^5^ cells/well) on 6-well plates and were allowed to grow in DMEM (supplemented with 10% heat-inactivated fetal bovine serum, 100 U/mL penicillin, 100 µg/mL streptomycin) for 48 h. Cells were then transfected with appropriate plasmids (3 µg PAD4 plasmid and 2 µg pIDTSMART eRF1-E55D for each well) using Lipofectamine 2000 (12.5 µg for each well) in Opti-MEM medium (Gibco). After 5 h, the medium was changed to DMEM and DMSO (for WT PAD4) or SM60 (1 mM for R374Cit PAD4) was added. Cells were then allowed to grow at 37 °C for 48 h. Cells were washed with serum-free DMEM (supplemented with 100 U/mL penicillin, 100 µg/mL streptomycin), resuspended in the same medium, and were irradiated with 365 nm UV for 3 min. Then, the cells were treated with CaCl_2_ (1 mM) and a combination of CaCl_2_ (1 mM) and ionomycin (5 µM) for 3 h at 37 °C. After this, cells were lysed and the lysates were analyzed by Western blotting using anti-histone H3 Cit (R2,8,17) and anti-histone H3 primary antibodies. PAD4 expression was quantified by using an anti-PAD4 primary antibody. Band intensities for citrullinated histone H3 were normalized against those for histone H3 and PAD4 using the following algorithm:

3 $${\mathrm{Normalized}}\,{\mathrm{histone}}\,{\mathrm{H3Cit}} = \left[ {{\mathrm{H3Cit}}/\left( {{\mathrm{H3}} \times {\mathrm{PAD4}}} \right)} \right] \times 10^6.$$

All the experiments were performed in triplicate. The full gels for the histone H3 citrullination by WT and R374Cit PAD4 are given in Supplementary Fig. 20c.

### Thermal shift assay

A 50 µL reaction mixture (EXPI293F lysate containing overexpressed WT PAD4 (1.5 mg/mL) or R374Cit mutant (2 mg/mL), 1× PBS and DMSO (1% final)) was heated at various temperatures (25, 40, 50, 60, 65, 70, 75, 85 and 90 °C) for 5 min, followed by flash freezing with liquid nitrogen. The samples were thawed and the precipitated proteins were separated by centrifugation (21,000 × *g*, 30 min) at 4 °C. Forty microliters of the supernatant was mixed with 10 µL of 5× SDS-PAGE loading dye and the mixture was boiled at 95 °C for 10 min. The proteins were separated by SDS-PAGE using a 4–20% gradient gel and the soluble fractions of PAD4 were quantitated by western blot analysis using a rabbit polyclonal anti-PAD4 antibody. This assay was also performed separately in the presence of CaCl_2_ (1 mM final) and a PAD4-selective ligand, GSK199 (10 µM final). For these assays, the reaction mixture was incubated with CaCl_2_ or GSK199 at room temperature for 5 min before heating up at different temperatures. All the reactions were performed at least in duplicate.

### Proteomics study on autocitrullinated PAD4

Fifty micrograms of PAD4 was incubated at 37 °C for various times (0, 5, 15, 30, and 90 min) in the absence and presence (10 mM) of CaCl_2_, followed by flash freezing with liquid nitrogen. The samples were thawed and trichloroacetic acid (20% final) was added. Then, the samples were placed at −20 °C for 30 min and the precipitated proteins were collected by centrifugation (21,000 × *g*, 30 min) at 4 °C, washed with cold acetone, and dried. The protein pellet was resuspended in 6 M urea (100 µL) in PBS and TCEP (tris(2-carboxyethyl)phosphine) (2 mM final) was added to it. The solution was incubated at 37 °C for 1 h. Then iodoacetamide (4 mM final) was added and the solution was incubated at 37 °C for 30 min in dark. Then, 200 µL of PBS was added to the solution to achieve a final concentration of urea of 2 M. Lys-C (1 µL of a 1 µg/µL solution, reconstituted in water) and Glu-C (2 µL of a 0.5 µg/µL solution, reconstituted in water) were then added to the samples and then incubated at 37 °C for 16 h. The proteolysis reaction was terminated by adding 15 µL of formic acid. The peptide mixture was then desalted with Pierce^TM^ desalting C18 spin columns, resuspended in 120 µL of HEPES buffer (100 mM, pH 8.5), and the total peptide content in each sample was quantified and normalized using the Pierce^TM^ Quantitative Fluorometric Peptide Assay kit, according to the manufacturer’s protocol. Eight microliters of a 19.5 µg/µL acetonitrile stock of TMT10plex^TM^ isobaric labeling reagents (TMT10-126, TMT10-127N, TMT10-127C, TMT10-128N, TMT10-128C, TMT10-129N, TMT10-129C, TMT10-130N, TMT10-130C, and TMT10-131) were added to 100 µL samples treated in the absence and presence of calcium for five different time points (0, 5, 15, 30, and 90 min). The reaction mixtures were incubated at room temperature for 1 h. To quench the reaction, 8 µL of 5% hydroxylamine was added to each sample and the mixture was incubated at room temperature for 15 min. Then, the ten samples (0, 5, 15, 30, and 90 min samples in the absence and presence of calcium) were combined together in a new microcentrifuge tube and desalted using C18 spin columns. This experiment was performed in triplicate.

### LC-MS/MS analysis

Peptides were lyophilized, resuspended in 5% acetonitrile, 0.1% (v/v) formic acid in water, and loaded at 4.0 µL/min by a NanoAcquity UPLC (Waters Corporation, Milford, MA) onto a 100 µm I.D. fused-silica pre-column packed with 2 cm of 5 µm (200 Å) Magic C18AQ (Bruker-Michrom), equilibrated with 5% acetonitrile, 0.1% (v/v) formic acid in water. After trapping for 4.0 min on the pre-column, peptides were eluted at 300 nL/min from a 75 µm I.D. gravity-pulled analytical column packed with 25 cm of 3 µm (100 Å) Magic C18AQ particles using a gradient of mobile phase A, 0.1% (v/v) formic acid in water and mobile phase B, 0.1% (v/v) formic acid in acetonitrile as follows: 0–100 min (5–35 %B), 100–120 min (35–65%B), 120–121 min (65–95 %B), and 121–126 min (95%B). Ions were introduced by positive ESI via liquid junction at 1.4 kV into a Q Exactive hybrid quadrapole orbitrap mass spectrometer (Thermo Scientific, Waltham, MA). Mass spectra were acquired over *m/z* 300–1750 at 70,000 resolution (*m/z* 200) with an AGC target of 1e6, and data-dependent acquisition selected the top 10 most abundant precursor ions for tandem MS by higher energy collisional dissociation fragmentation using an isolation width of 1.6 Da, max fill time of 100 ms, and AGC target of 1e5. Peptides were fragmented by a normalized collisional energy (NCE) of 27 and product ion spectra acquired at a resolution of 17,500 (*m/z* 200). For TMT-labeled samples, NCE was set to 32 and product ion spectra were acquired at a resolution of 35,000 (*m/z* 200).

### LC-MS/MS data analysis

Raw data files were peak processed with Proteome Discoverer (version 2.1, Thermo Scientific, Waltham, MA), followed by identification using Mascot Server (version 2.5, Matrix Science) against the Swissprot human or *E. coli* (TMT-labeled samples) FASTA file. Proteolytic enzyme was set to Lys-C and Glu-C with two missed cleavages. Variable modifications of N-terminal acetylation, oxidized methionine, pyroglutamic acid for glutamine, deamidation of asparagine, and citrullination of arginine were implemented. Carbamidomethylation of cysteines and TMT10-plex modification at lysine and peptide N terminus were set as fixed modifications. Assignments were made using a 10 p.p.m. mass tolerance for the precursor and 0.05 Da mass tolerance for the fragments. All non-filtered search results were processed by Scaffold (version 4.10.0, Proteome Software, Inc.) utilizing the Trans-Proteomic Pipeline (Institute for Systems Biology) with threshold values set at 90% for peptides (1% false-discovery rate) and 99% for proteins (two peptides minimum, 6% false-discovery rate). TMT product ion ratios were calculated using the Scaffold Q + S analysis software.

### Statistics and reproducibility

Experiments corresponding to Figs. 2a, c and 3b, c and Supplementary Fig. 7 were independently repeated three times with similar results. Experiments corresponding to Figs. 2d and 3a and Supplementary Figs. 3–6, 8–9, 14, 16b, c, 17a–f, and 18 were independently repeated two times with similar results.

### Biological materials

All the plasmids are available upon request to the corresponding authors (P.R.T. and A.C.) by email.

### Reporting summary

Further information on research design is available in the Nature Research Reporting Summary linked to this article.

## Supplementary information

Supplementary Information

Reporting Summary

Peer Review File

## Data Availability

All the raw data are available upon request to the corresponding authors (P.R.T. and A.C.) by email. Source data are provided with this paper.

## Source data

Source Data
