# Supplementary material for: Site-specific incorporation of citrulline into proteins in mammalian cells
Source: Nat Commun. 2021 Jan 4;12:45. doi: 10.1038/s41467-020-20279-w (PMC7782748; doi:10.1038/s41467-020-20279-w)
Supplement: Supplementary file 1 — Supplementary Information [file 41467_2020_20279_MOESM1_ESM.pdf]

Supplementary information for:

Site-Specific Incorporation of Citrulline into Proteins in Mammalian Cells

Santanu Mondal,[a][c] Shu Wang,[b][c] Yunan Zheng,[b] Sudeshna Sen,[a] Abhishek Chatterjee*,[b] and Paul R. Thompson*[a]

[a]Department of Biochemistry and Molecular Pharmacology, UMass Medical School, 364 Plantation Street, Worcester, MA 01605, USA

[b]Department of Chemistry, Boston College, Chestnut Hill, Massachusetts 02467, USA

[c]These authors contributed equally to this work.

*Author to whom correspondence should be addressed: Department of Biochemistry and Molecular Pharmacology, University of Massachusetts Medical School, LRB 826, 364 Plantation Street, Worcester MA 01605, USA, tel: 508-856-8492, fax: 508-856-6215; Department of Chemistry, Boston College, Chestnut Hill, MA 02467, USA, tel: 617-552-1778, fax: 617-552-2705

e-mail: paul.thompson@umassmed.edu, abhishek.chatterjee@bc.edu

Supplementary Table 1. Sites of autocitrullination in PAD4 and peptides from which they are detected by tandem mass spectrometry.

Site	Peptide	Protease Used	Reference
R123	ISLCADITRTGK	Glu-C/Lys-C	This study
	ALLYLTAVEISLCADITRTGK	Trypsin	1[a]
	ALLYLTAVEISLCADITR	Trypsin	1[b]
R156	TWTWGPCGQGAILLVNCDR	Trypsin	1[b]
R205	DFFTNHTLVLHVAR	Trypsin	1[b]
	DFFTNHTLVLHVARSEMDK	Trypsin	2
R212/218	MDKVRVFQATRGK	Glu-C/Lys-C	This study
	VRVFQATRGK	Trypsin	2
R218	VFQATRGK	Trypsin	2
R372	TLPVVFDSPRNRGLK	Glu-C/Lys-C	This study
R374	TLPVVFDSPRNRGLKE	Glu-C/Lys-C	This study
R372/374	TLPVVFDSPRNRGLKE	Glu-C/Lys-C	This study
	TLPVVFDSPRNRGLK	Trypsin	2
R383	RVMGPDFGYVTR	Trypsin	2
R394	RVMGPDFGYVTRGPQTGGISGLDSFGNLE	Glu-C/Lys-C	This study
	VMGPDFGYVTR	Trypsin	2
R419	VSPPVTVRGK	Glu-C/Lys-C	This study
	GPQTGGISGLDSFGNLEVSPPVTVR	Trypsin	1[c]
R484	FLSFVPAPDRK	Glu-C/Lys-C	This study
	LYSDWLSVGHVDEFLSFVPAPDR	Trypsin	1[b]
R484/488/495	FLSFVPAPDRKGFRLLLASPRSCYK	Glu-C/Lys-C	This study
R488/R495	GFRLLLASPRSCYK	Glu-C/Lys-C	This study
R495	GFRLLLASPRSCYK	Glu-C/Lys-C	This study
	LLLASPRSCYK	Trypsin	2
R536	NILSNKTLRE	Glu-C/Lys-C	This study
R544	HNSFVERCIDWNRE	Glu-C/Lys-C	This study
R536/R544	TLREHNSFVER	Trypsin	2
R609	HLGIPKPFGPVINGR	Trypsin	1[a]
R639	VCSLLEPLGLQCTFINDFFTYHIR	Trypsin	1[b]
R650/651	VHCGTNVRRKPFSFK	Glu-C/Lys-C	This study

[a]Detected from endogenous PAD4 in HL-60 cells. [b]Detected from recombinant PAD4 reconstituted in ^{18}O labeled water. [c]Detected from recombinant PAD4 reconstituted in normal water.

Supplementary Table 2. Ions Detected from LC-MS/MS Analysis of R372Cit PAD4 digested with Lys-C and Glu-C.

Peptide sequence	Charge	Observed Mass	Actual Mass
^{61}KSTGSSTWPLDPGVE^{75}	2	780.88	1,559.75
^{62}STGSSTWPLDPGVE^{75}	2	716.83	1,431.65
^{92}VQISYYGPKTPPVK^{105}	3	526.30	1,575.87
^{115}ISLCADITRTGK^{126}	3	445.57	1,333.70[*]
^{180}MSLMTLSTKTPK^{191}	3	446.58	1,336.71
^{192}DFFTNHTLVLHVARSE^{207}	3	629.32	1884.95
^{192}DFFTNHTLVLHVARSE^{207}	4	472.24	1,884.95
^{288}SVVFRVAPWIMTPNTQPPQE^{307}	2	1149.09	2,296.16
	3	766.40	2,296.16
	3	771.73	2312.16[†]
^{318}DFLKSVTTLAMK^{329}	2	677.38	1,352.74
	3	451.92	1,352.74
^{354}IGYIQAPHK^{362}	2	513.79	1,025.57
	3	342.86	1,025.57
^{354}IGYIQAPHKTLPVVFD^{369}	2	899.50	1,796.98
	3	600.00	1,796.98
^{354}IGYIQAPHKTLPVVFDSPRNRGLK^{377}	5	542.31	2706.50[ǁ]
^{363}TLPVVFDSPRNRGLK^{377}	3	567.32	1,698.94[***]
^{363}TLPVVFDSPRNRGLK^{377}	3	610.34	1,827.98[ǁ]
^{379}FPIKRVMGPD^{388}	3	387.21	1,158.62
^{383}RVMGPDFGYVTRGPQTGGISGLDSFGNLE^{411}	3	1,009.83	3,026.45
^{383}RVMGPDFGYVTRGPQTGGISGLDSFGNLE^{411}	3	1,015.16	3,042.45[†]
^{389}FGYVTRGPQTGGISGLDSFGNLE^{411}	3	791.40	2,371.16
^{440}SRQMHQALQDFLSAQQVQAPVK^{461}	4	628.33	2,509.29
^{450}FLSAQQVQAPVK^{461}	2	658.37	1,314.73
^{462}LYSDWLSVGHVDE^{474}	2	760.36	1,518.70
	3	507.24	1,518.70
^{475}FLSFVPAPDRK^{485}	2	638.86	1,275.70
	3	426.24	1,275.70
^{486}GFRLLLASPRSCYK^{499}	3	556.64	1,666.90[*]
	4	417.73	1,666.90[*]
^{508}GHGEALLFE^{516}	2	486.74	971.47
^{512}ALLFEGIK^{519}	2	445.77	889.53
^{526}IKNILSNK^{533}	3	310.53	928.57
^{526}IKNILSNKTLRE^{537}	2	714.93	1,427.84
	3	476.96	1,427.84
	4	357.97	1,427.84
^{528}NILSNKTLRE^{537}	3	396.56	1,186.67
	3	396.89	1,187.65[ǁ]
	2	594.34	1,186.67
^{562}SDIIDIPQLFK^{572}	2	644.86	1,287.71
^{581}AFFPNMVNMLVLGK^{594}	2	790.92	1,579.82

	3	527.62	1,579.82
[581]AFFPNMVNMLVLGKHLGIPKPFGPVINGRCCLE[613]	5	745.99	3,724.91*
	5	746.59	3,727.91**
[595]HLGIPKPFGPVINGRCCLE[613]	3	722.04	2,163.11*
	3	722.37	2,164.09**
[615]KVCSLLEPLGLQCTFIND[632]	2	1,054.03	2,106.05*
	3	703.02	2,106.05*
[633]FFTYHIRHGE[642]	2	653.82	1,305.63
	3	436.22	1,305.63
	4	327.41	1,305.62

* Modifications included: carbamidomethylation
** Modifications included: carbamidomethylation, deamidation
*** Modifications included: citrullination
† Modifications included: oxidation
ǀ Modifications included: deamidation

Supplementary Table 3. Ions Detected from LC-MS/MS Analysis of R374Cit PAD4 digested with Lys-C and Glu-C.			
Peptide sequence	Charge	Observed Mass	Actual Mass
^{61}KSTGSSTWPLDPGVE^{75}	2	780.8801	1,559.75
	3	520.9243	1,559.75
^{62}STGSSTWPLDPGVE^{75}	2	716.8336	1,431.65
^{92}VQISYYGPKTPPVK^{105}	3	526.2959	1,575.87
^{92}VQISYYGPKTPPVKALLYLTAVE^{114}	3	850.8123	2,549.41
^{106}ALLYLTAVE^{114}	2	496.7865	991.5584
^{115}ISLCADITRTGK^{126}	2	667.858	1,333.70*
	3	445.5746	1,333.70*
^{180}MSLMTLSTKTPK^{191}	3	446.5774	1,336.71
^{192}DFFTNHTLVLHVARSE^{207}	4	472.2447	1,884.95
^{225}CSVVLGPKWPSHYLMVPGGK^{244}	4	553.7913	2,211.14*
^{245}HNMDFYVE^{252}	2	527.7189	1,053.42
^{253}ALAFPDTDFPGLITLTISLLD^{273}	2	1,117.10	2,232.19
	3	745.0708	2,232.19
^{282}AVVFQDSVVFRVAPWIMTPNTQPPQE^{307}	3	986.1743	2,955.50
	4	739.8812	2,955.50
^{288}SVVFRVAPWIMTPNTQPPQE^{307}	2	1,149.09	2,296.16
	3	766.3973	2,296.16
	4	575.0493	2,296.16
^{288}SVVFRVAPWIMTPNTQPPQEVYACSIFE^{315}	3	1,089.87	3,266.58**
^{308}VYACSIFE^{315}	2	494.7257	987.4369*
^{318}DFLKSVTTLAMK^{329}	2	677.3763	1,352.74
	3	451.9195	1,352.74
^{354}IGYIQAPHK^{362}	3	342.8627	1,025.57
^{354}IGYIQAPHKTLPVVFD^{369}	2	899.4986	1,796.98
	3	600.001	1,796.98
^{363}TLPVVFD^{369}	2	395.7211	789.4276
^{363}TLPVVFDSPRNRGLK^{377}	3	567.3207	1,698.94***
^{370}SPRNRGLKEFPIK^{382}	4	386.4741	1,541.87***
^{383}RVMGPDFGYVTRGPQTGGISGLDSFGNLE^{411}	3	1,009.83	3,026.46
	4	757.6212	3,026.46
^{389}FGYVTRGPQTGGISGLDSFGNLE^{411}	2	1,186.58	2,371.15
	3	791.3882	2,371.15
^{440}SRQMHQALQDFLSAQQVQAPVK^{461}	4	628.3287	2,509.29
^{450}FLSAQQVQAPVK^{461}	2	658.371	1,314.73
^{462}LYSDWLSVGHVDE^{474}	2	760.3563	1,518.70
	3	507.2402	1,518.70
^{475}FLSFVPAPDRK^{485}	2	638.856	1,275.70
	3	426.2394	1,275.70
^{486}GFRLLLASPRSCYK^{499}	3	556.639	1,666.90*
	4	417.7319	1,666.90*
^{508}GHGEALLFE^{516}	2	486.743	971.4714
^{512}ALLFEGIK^{519}	2	445.7715	889.5285

^{526}IKNILSNKTLRE^{537}	2	714.9295	1,427.84
	3	476.9563	1,427.84
	4	357.9686	1,427.84
^{528}NILSNKTLRE^{537}	2	594.3407	1,186.67
	3	396.5632	1,186.67
^{538}HNSFVERCIDWNRE^{551}	3	621.2845	1,860.83*
	4	466.2158	1,860.83*
^{562}SDIIDIPQLFK^{572}	2	644.8606	1,287.71
	3	430.2436	1,287.71
^{579}AEAFFPNMVNMLVLGK^{594}	2	890.9604	1,779.91
	3	594.3103	1,779.91
^{581}AFFPNMVNMLVLGK^{594}	2	790.9189	1,579.82
	3	527.6149	1,579.82
^{581}AFFPNMVNMLVLGKHLGIPKPFGPVINGRCCLE^{613}	5	745.99	3,724.91*
^{595}HLGIPKPFGPVINGRCCLE^{613}	3	722.0433	2,163.11*
^{615}KVCSLLEPLGLQCTFIND^{632}	2	1,054.03	2,106.05*
	3	703.023	2,106.05*
^{616}VCSLLEPLGLQCTFIND^{632}	3	660.326	1,977.96*
^{616}VCSLLEPLGLQCTFINDFFTYHIRHGE^{642}	4	817.8986	3,267.57**
^{633}FFTYHIRHGE^{642}	2	653.8193	1,305.62
	3	436.2157	1,305.62
	4	327.4137	1,305.62

* Modifications included: carbamidomethylation
** Modifications included: carbamidomethylation, deamidation
*** Modifications included: citrullination

Supplementary Table 4. Steady-state kinetic parameters for wild-type $PAD4_{Bac}$, wild-type $PAD4_{Mam}$, R372Cit and R374Cit mutants.

Enzyme	k_{cat} (S^{-1})	K_m (mM)	k_{cat}/K_m ($M^{-1}S^{-1}$)	$K_{0.5}$ (mM)
$PAD4_{Bac}$	3.4 ± 0.2	0.7 ± 0.1	4740 ± 260	0.9 ± 0.10
$PAD4_{Mam}$	3.7 ± 0.1	1.1 ± 0.2	3620 ± 810	0.9 ± 0.04
R372Cit	-	-	20 ± 2	-
R374Cit	0.33 ± 0.01	0.84 ± 0.06	390 ± 40	-

k_{cat}: turnover number; K_m: Michaelis-Menten constant; k_{cat}/K_m: catalytic efficiency; $K_{0.5}$: Ca^{2+} concentration for half-maximal activity.

Supplementary Table 5. Normalized† fold changes of the peptides containing Arg and Cit at various autocitrullination sites with increasing time in the absence and presence of calcium.

Site	Status	$CaCl_2$ (0 mM)					$CaCl_2$ (10 mM)				
		0 min	5 min	15 min	30 min	90 min	0 min	5 min	15 min	30 min	90 min
123	Arg	1	1.060198	1.099362	1.086233	0.847332	1	1.015601	1.108801	0.955283	0.90346
	Cit	1	1.111622	0.681444	1.061178	0.931525	1	5.35171	7.061624	6.900336	7.226682
212/218	Arg	1	0.763923	0.717972	0.867539	0.447513	1	0.844928	1.023137	1.030968	0.845279
	Cit	1	1.043454	0.542113	0.587774	0.614152	1	8.693879	13.17746	12.295	14.45336
372	Arg	1	0.883519	1.368884	0.993322	0.918658	1	1.112136	1.391525	1.316463	1.254982
	Cit	1	1.176907	0.858565	0.584389	1.82134	1	3.093736	4.228072	5.637283	6.04189
374	Arg	1	0.875998	0.849096	0.846745	0.939523	1	0.869947	0.809442	1.046085	0.90485
	Cit	1	1.095559	0.741919	0.930278	1.176091	1	4.169863	4.267329	6.175974	7.094331
372/374	Arg	1	0.875998	0.849096	0.846745	0.939523	1	0.869947	0.809442	1.046085	0.90485
	Cit	1	1.294953	1.149787	0.90417	1.006956	1	4.018527	4.30695	5.81589	6.233317
394	Arg	1	0.901459	1.284909	1.065847	1.397647	1	0.942636	0.987145	0.965936	0.99424
	Cit	1	1.107009	1.01607	1.156688	1.256723	1	1.528377	1.672493	1.699763	1.930088
419	Arg	1	0.827406	1.071773	0.860154	0.609628	1	1.271619	1.311302	1.23086	1.387031
	Cit	1	0.832199	0.72951	0.803851	0.869545	1	2.361985	2.763826	2.367449	2.578741
484	Arg	1	0.92959	1.047052	0.926588	0.899794	1	1.242001	1.414214	1.310393	1.420436
	Cit	1	1.138131	0.573024	0.833161	1.173919	1	9.210844	11.60494	10.33882	11.87619
484/488/495	Cit	1	1.123889	0.467056	0.470848	0.52912	1	13.48546	16.22335	17.79418	19.97329
495	Arg	1	0.95418	1.118062	1.007654	0.982457	1	1.381913	1.689582	1.505247	1.691262
	Cit	1	0.903753	0.697533	0.91088	1.074749	1	2.757447	3.363586	3.294364	3.547166
488/495	Arg	1	0.95418	1.118062	1.007654	0.982457	1	1.381913	1.689582	1.505247	1.691262
	Cit	1	0.972655	0.82932	0.768438	1.170128	1	2.909961	4.05865	4.153517	4.771138
536	Arg	1	0.965267	0.835088	1.035026	0.652176	1	0.932817	0.970859	0.828171	0.870752
	Cit	1	0.808321	0.960595	0.93045	0.872363	1	4.808432	6.058245	5.274009	6.644835
544	Arg	1	0.997692	0.963707	0.905216	0.692555	1	0.960062	0.872081	1.013397	1.022755
	Cit	1	0.924236	0.929161	0.844401	0.875796	1	1.113087	1.076738	1.301342	1.410298
650/651	Arg	1	0.872161	0.893992	1.115739	0.769148	1	1.148698	1.202191	1.109467	1.248331
	Cit	1	1.038139	0.379893	0.671116	0.728681	1	4.505436	6.083915	6.520579	8.805063

†Ratios for the calcium- treated and untreated data set were normalized against the respective 0 min time-point.

Supplementary Table 6. List of Primers	
Name	Sequence
PAD4WT-Forward	ATTATTAGAATTGGCCAAGGAGGCCACCATGGACTACAAGG ACGACGACGACAAG
PAD4WT-Reverse	ATTATTAGAATTCGGCCTTAGAGGCCTCAGTGGTGGTGGTG GTGGTGGTGGTGGTGGTGGTGGGGCACCATGTTCCACC ACTTGAA
PAD4 R372TAG Inner Forward	GTCTTCGACTCTCCTTAGAACAGAGGCCTGAAG
PAD4 R372TAG Inner Reverse	CCTTCAGGCCTCTGTTCTAAGGAGAGTCGAAGAC
PAD4 R374TAG Inner Forward	GTCTTCGACTCTCCTAGGAACTAGGGCCTGAAG
PAD4 R374TAG Inner Reverse	CTTCAGGCCCTAGTTCCTAGGAGAGTCGAAGAC
eRF1-Forward	GCTAGCGCCGCCACCATGGCCGATGATCCAAGCGCCGCAG
eRF1-Reverse	ACTCGAGCTATCATTAGTAATCATCCAAATCGAAGAATTCATCATCTCCA

a

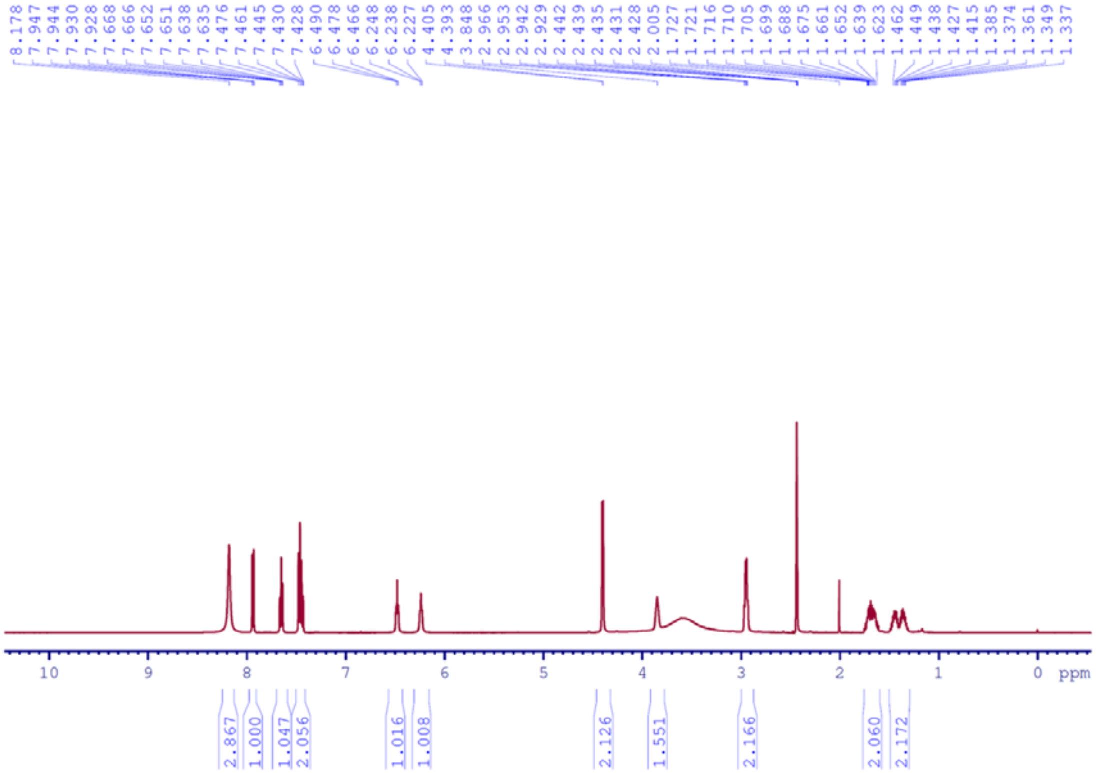

b

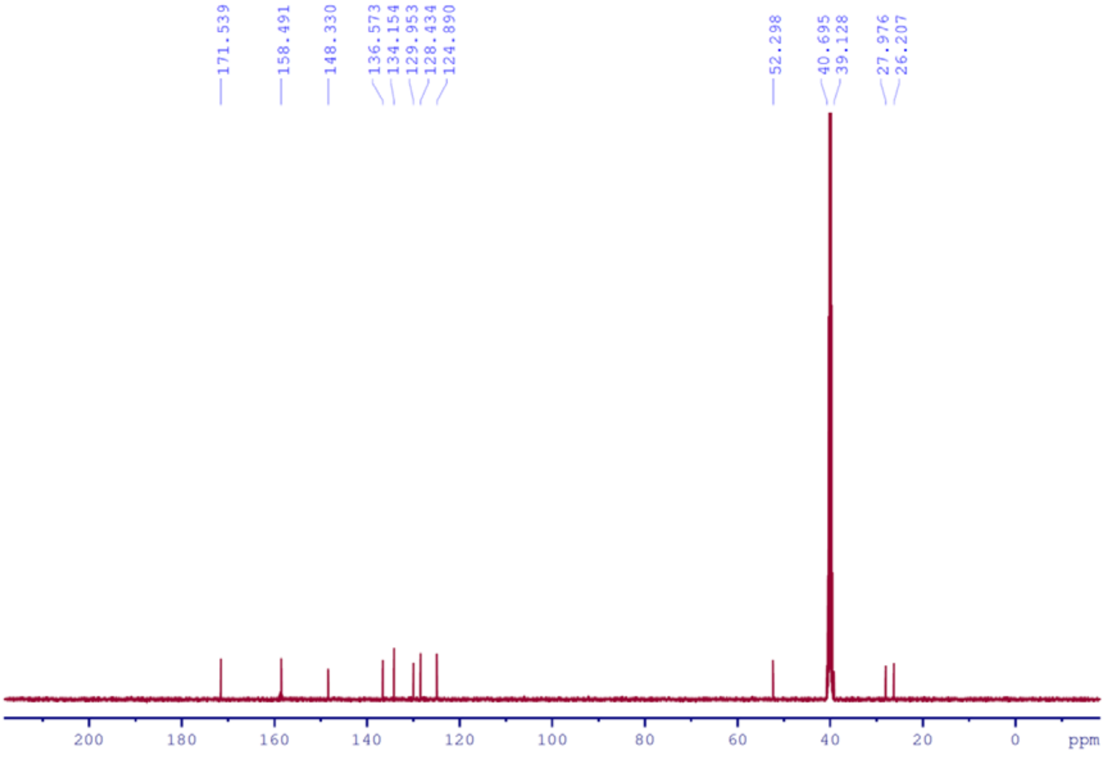

Supplementary Figure 1. ^{1}H (**a**) and ^{13}C (**b**) NMR of SM60 in DMSO-d_6.

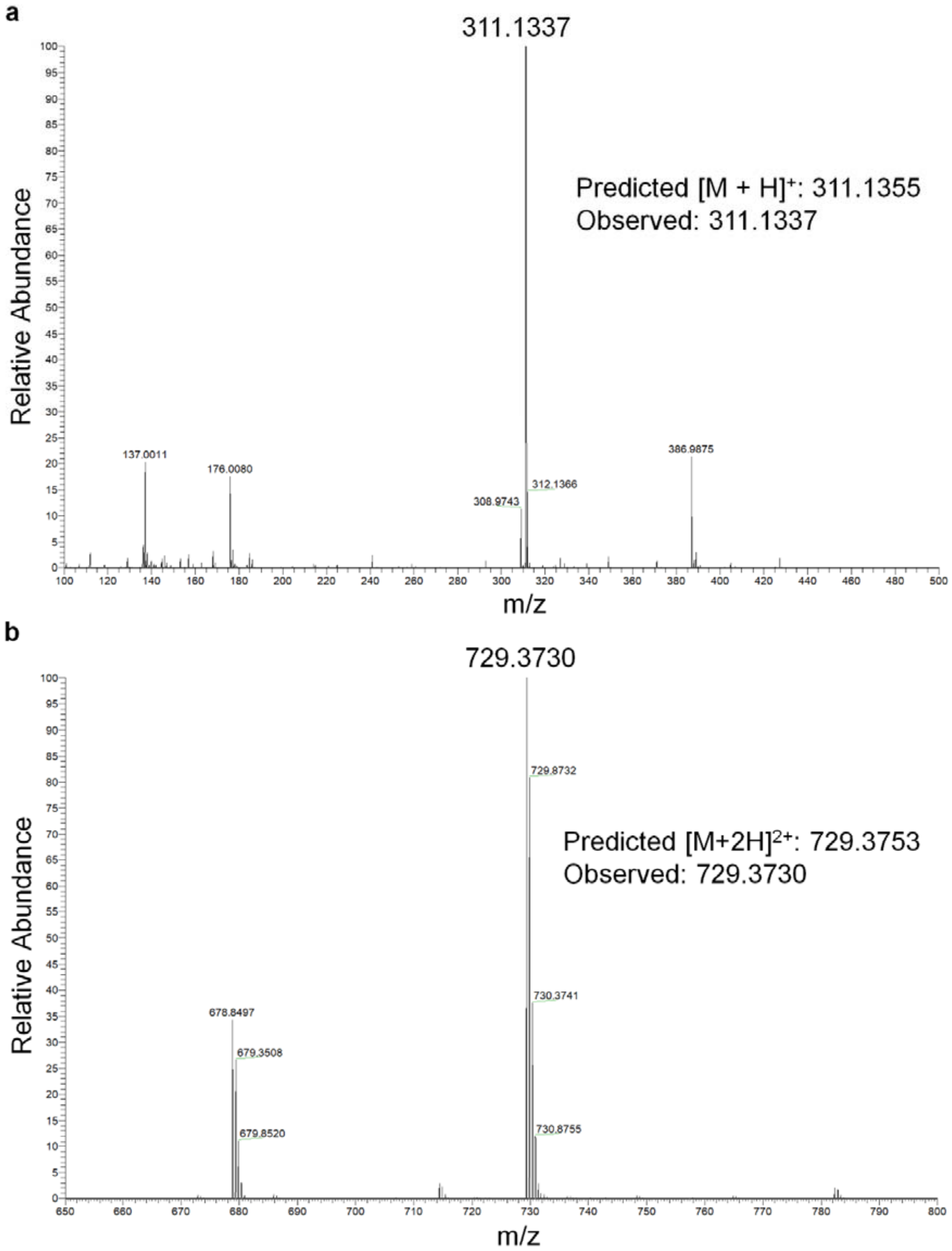

Supplementary Figure 2. High Resolution Mass Spectrum (HRMS) of SM60 (**a**) and SM70 (**b**).

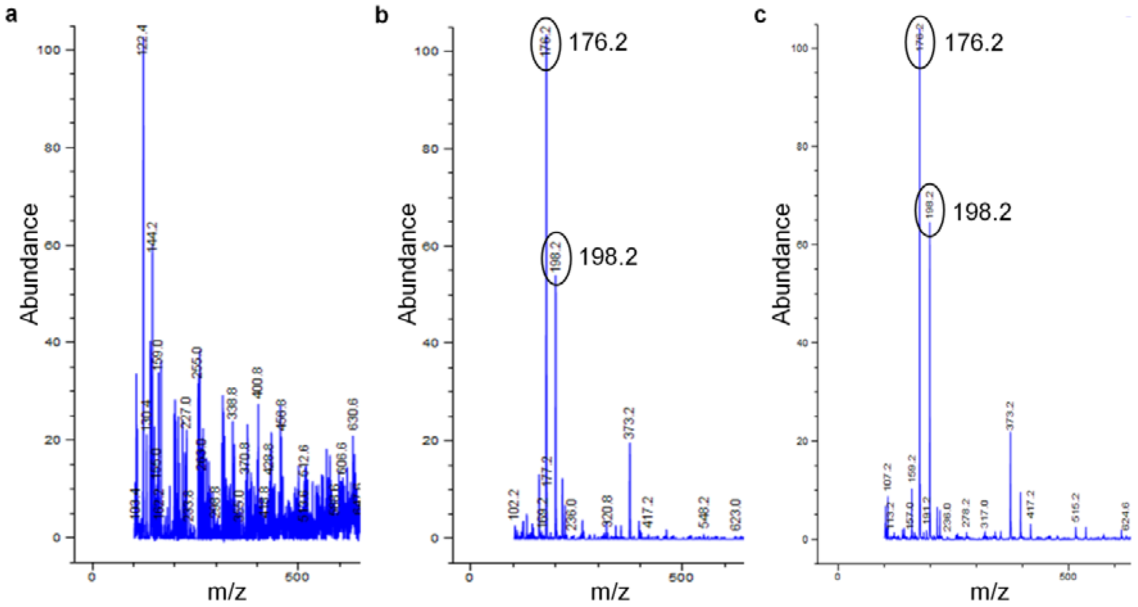

Supplementary Figure 3. ESI mass spectrum for the formation of citrulline from SM60 upon UV exposure. Citrulline appears at ~0.6 min in the ion chromatogram (Fig. 1**d**). While the peaks for citrulline are absent before UV exposure (**a**), they appear upon UV exposure and reach a maximum after 5 min treatment. The representative mass spectrum after 5 min exposure is shown in panel **b**. **c**, ESI mass spectrum of a standard sample of citrulline. ESI-MS (m/z) calculated for $C_6H_{13}N_3O_3$ $[M + H]^+$: 176.10, found 176.20; $[M + Na]^+$: 198.09, found 198.20.

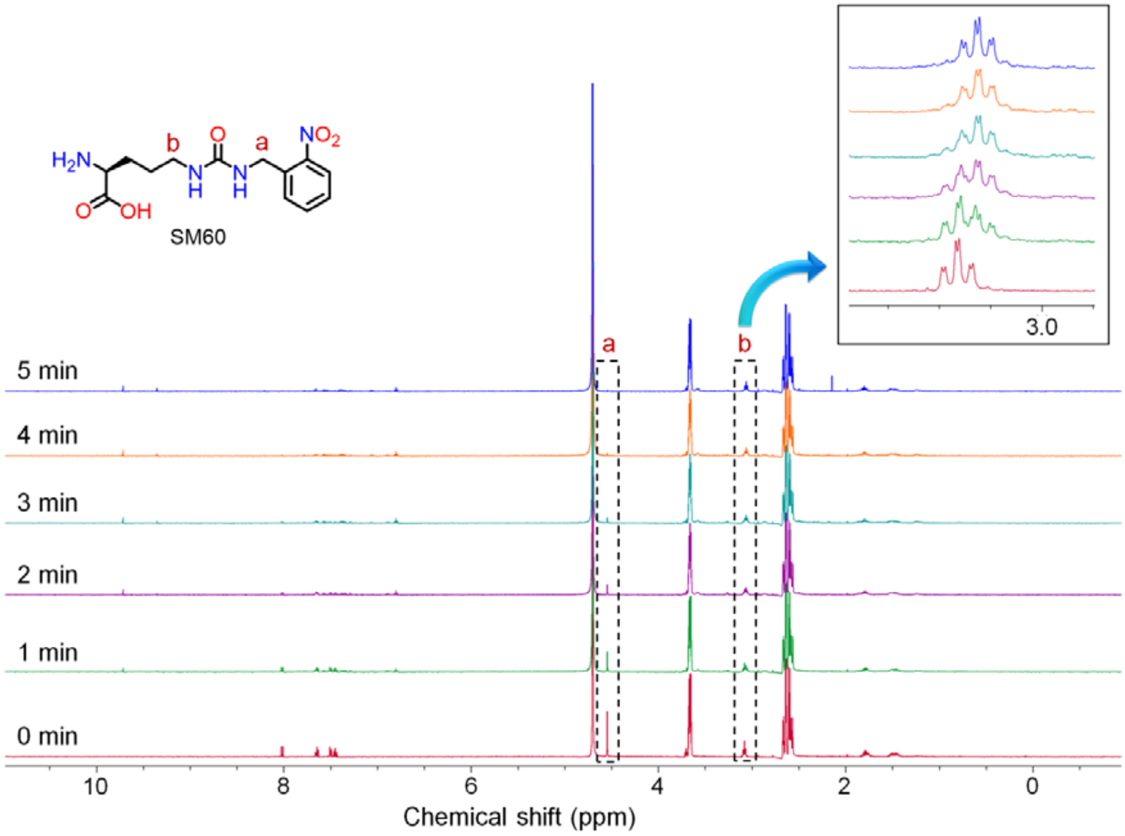

Supplementary Figure 4. ^{1}H NMR spectrum of SM60 in D_2O after UV (365 nm) exposure for 1, 2, 3, 4 and 5 min. The disappearance of the benzylic 'a' protons at 4.5 ppm (as seen in the spectrum denoted by 0 min) with increasing UV exposure indicates the complete removal of the *o*-nitrobenzyl photocage. Furthermore, an upfield shift was also observed for the 'b' protons upon the formation of citrulline. The multiplets at 3.6-3.7 and 2.6-2.7 ppm are due to DTT used in the assay. Assay condition: 1 mM SM60, 2 mM DTT, D_2O.

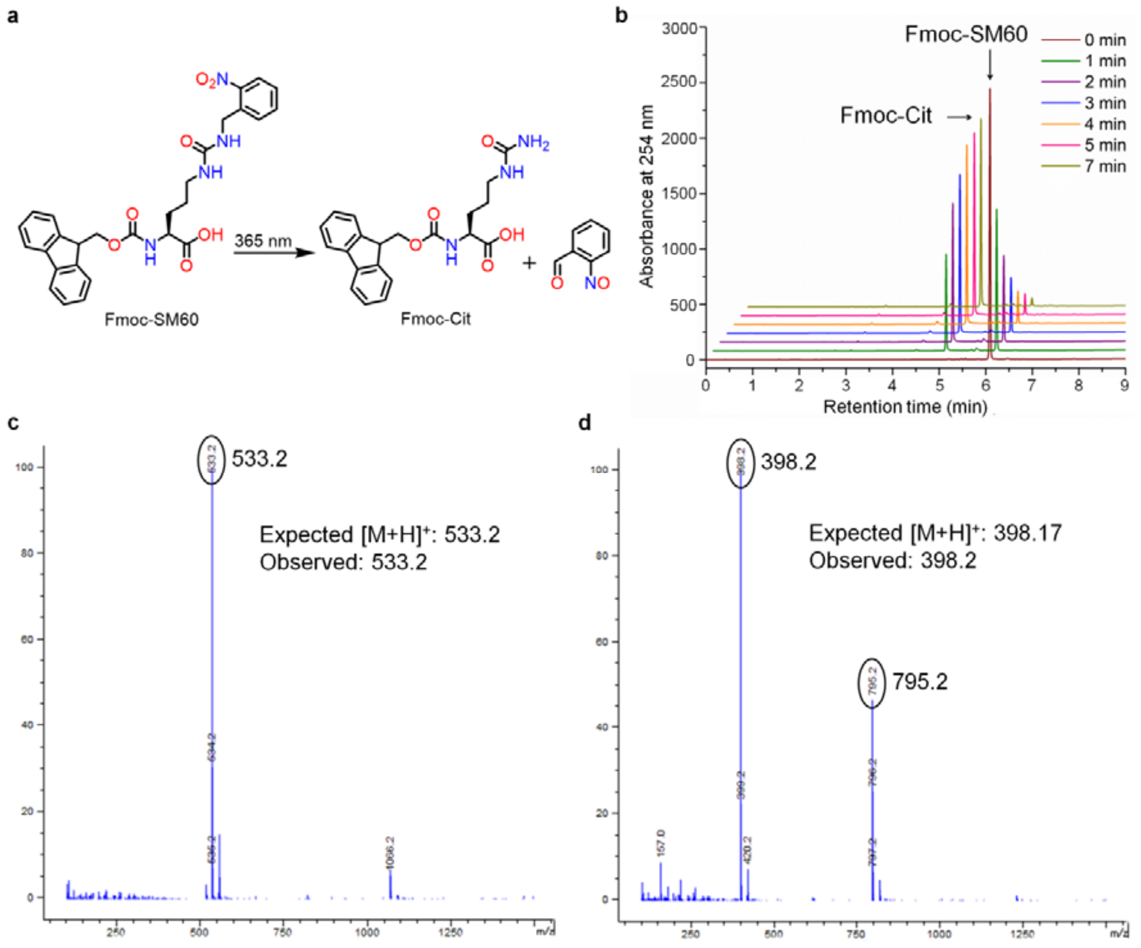

Supplementary Figure 5. **a**, Photodecaging of Fmoc-SM60 to produce Fmoc-Cit. **b**, HPLC chromatograms indicating the disappearance of Fmoc-SM60 and the production of Fmoc-Cit with increasing UV exposure. These chromatograms also indicate the quantitative conversion of Fmoc-SM60 to Fmoc-Cit. Assay mixture: 0.5 mM Fmoc-SM60, 2 mM DTT, Phosphate-buffered saline pH 7.4. ESI-Mass spectra of Fmoc-SM60 (**c**) and Fmoc-Cit (**d**).

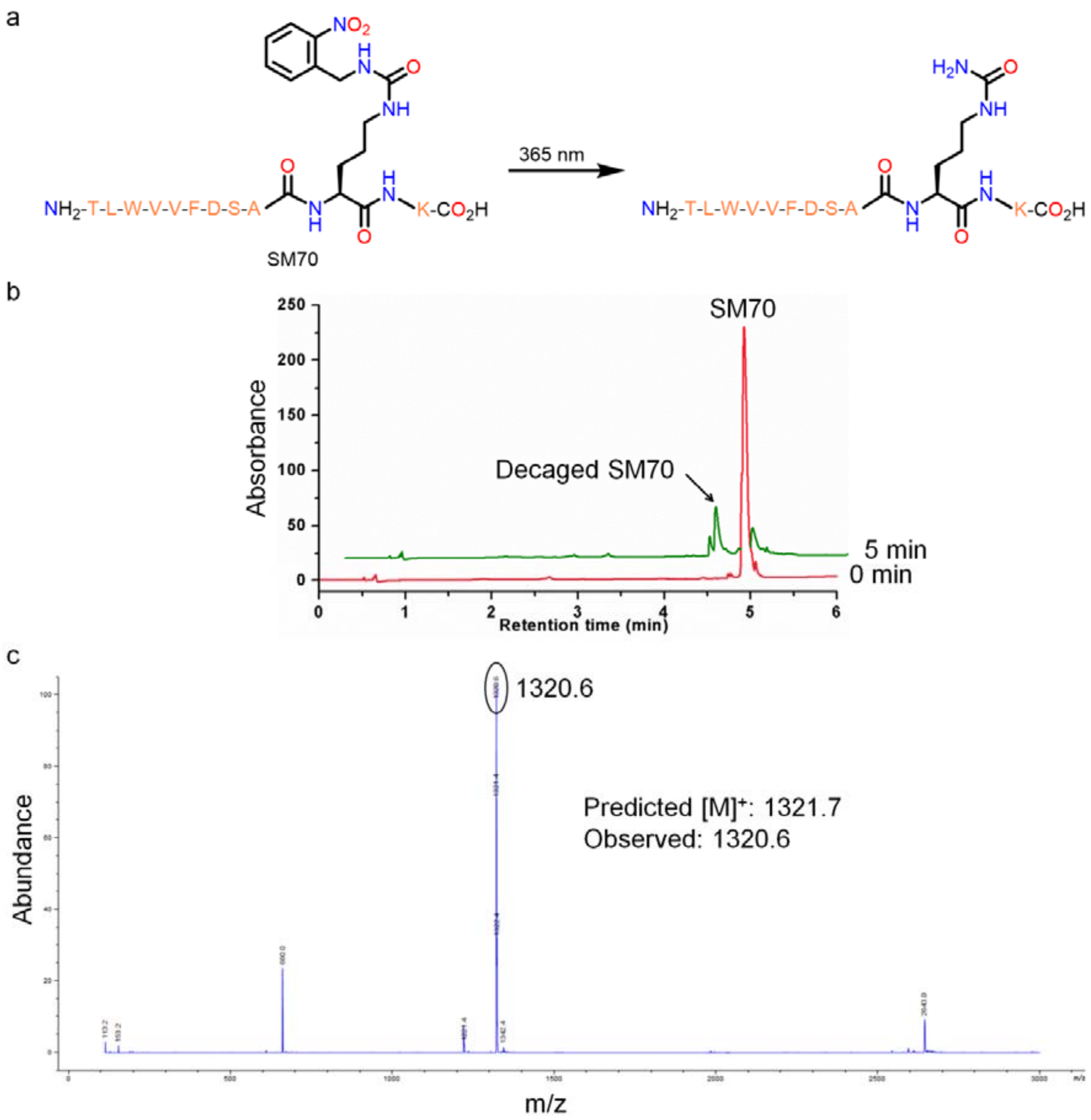

Supplementary Figure 6. **a**, Decaging of SM70, a PAD4-derived peptide (residues 363-372 with SM60 at the 372 position. Prolines at 365 and 371 positions were replaced with W and A, respectively, and a lysine residue was added to the C-terminus for ease of synthesis). **b**, HPLC chromatogram of SM70 before and after 5 min of 365 nm irradiation. The low peak intensity of the decaged product is explained by the lack of an aromatic group and its extremely low solubility in aqueous buffer. Even after performing the reaction in 1:1 acetonitrile/PBS, the solution became turbid upon the formation of the decaged peptide.

Assay condition: 200 μM SM70, 2 mM DTT, 1:1 acetonitrile/PBS (pH 7.4). **c**, ESI mass spectrum of the citrulline-containing peptide (decaged product).

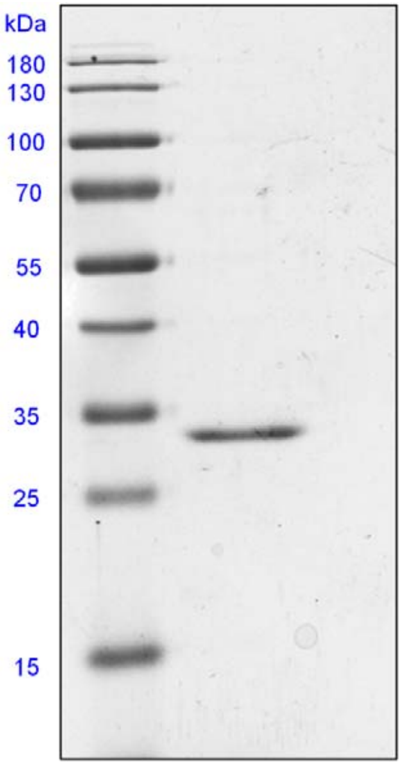

Supplementary Figure 7. Full gel for the Coomassie stain (corresponding to Fig. 2**c**) of purified EGFP containing **SM60** at 39 position.

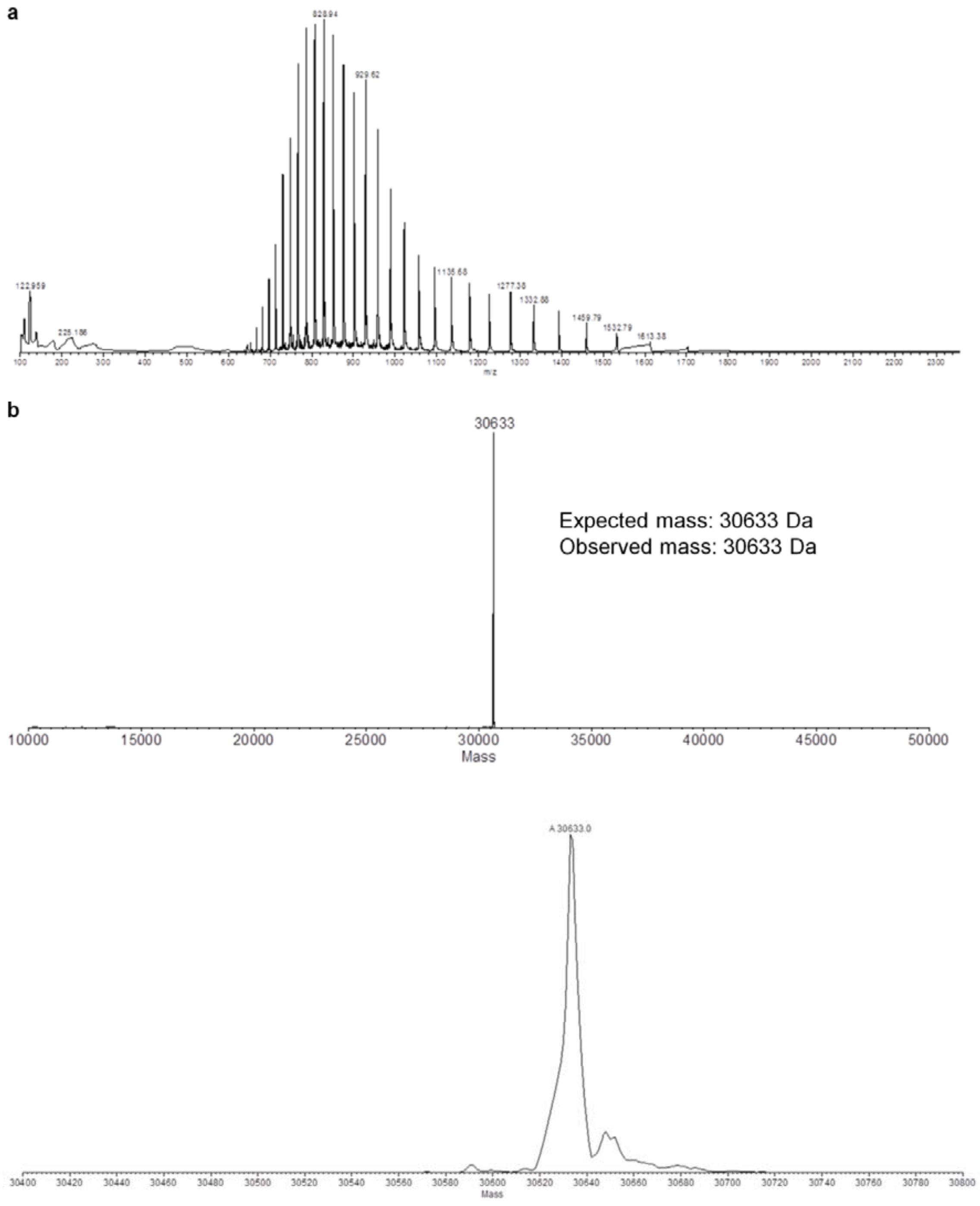

Supplementary Figure 8. ESI mass spectrum (**a**) and deconvoluted spectra (**b**) of EGFP containing SM60 at 39 position.

a

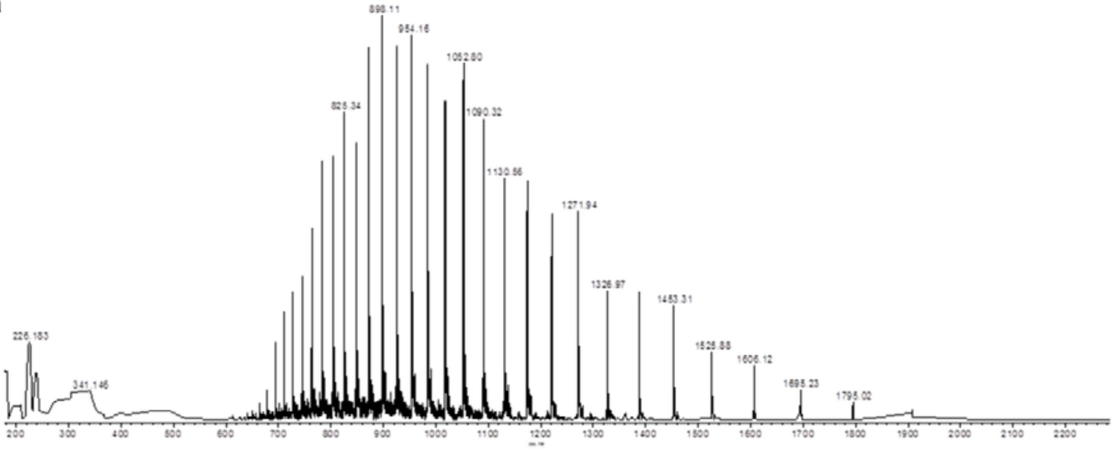

b

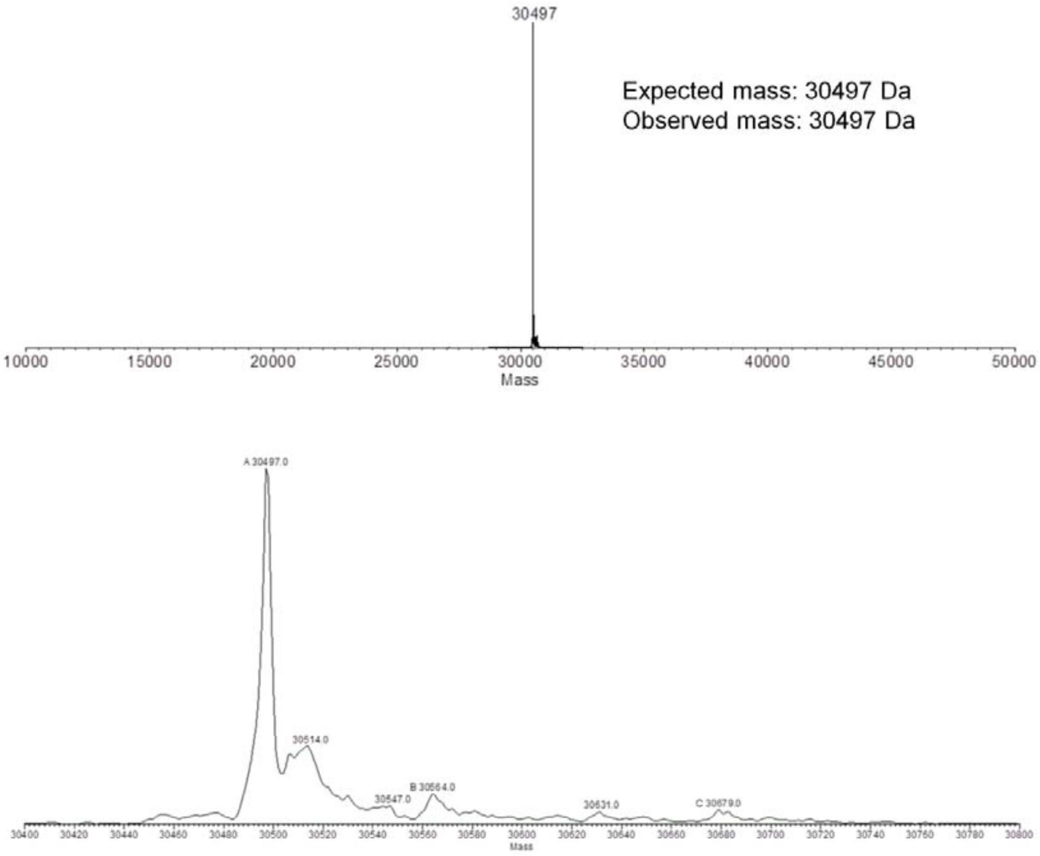

Supplementary Figure 9. ESI mass spectrum (**a**) and deconvoluted spectra (**b**) of EGFP containing **Cit** at 39 position.

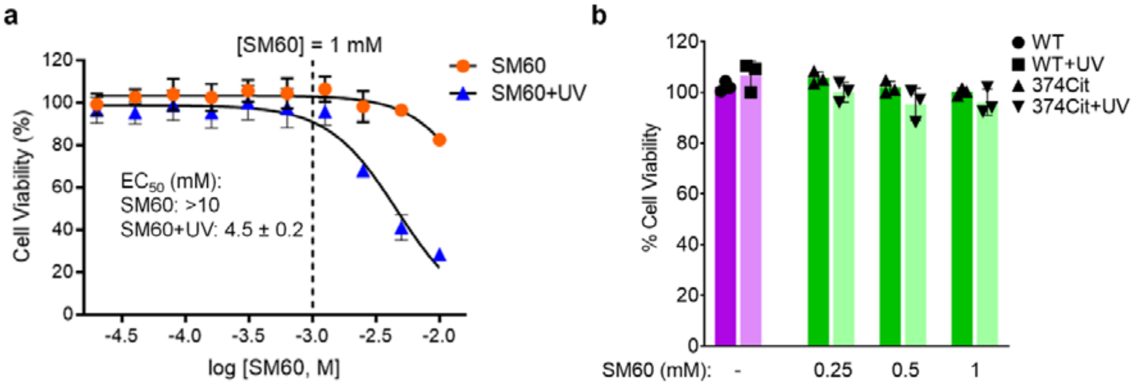

Supplementary Figure 10. **a**, Viability of HEK293T cells treated with increasing concentrations of SM60, and a combination of SM60 and 365 nm UV. The dotted line indicates the concentration (1 mM) used for citrulline incorporation in GFP and PAD4. **b**, Viability of HEK293T cells overexpressing WT and R374Cit PAD4 before and after photodecaging. These results indicate that PAD4 expression as well as light treatment does not cause cell death. Various concentrations of SM60 were used to induce increasing amounts of R374Cit mutant expression. For both the panels, n=3 independent experiments and data are presented as mean value ± SD. Source data for both panels are provided in the Source Data file.

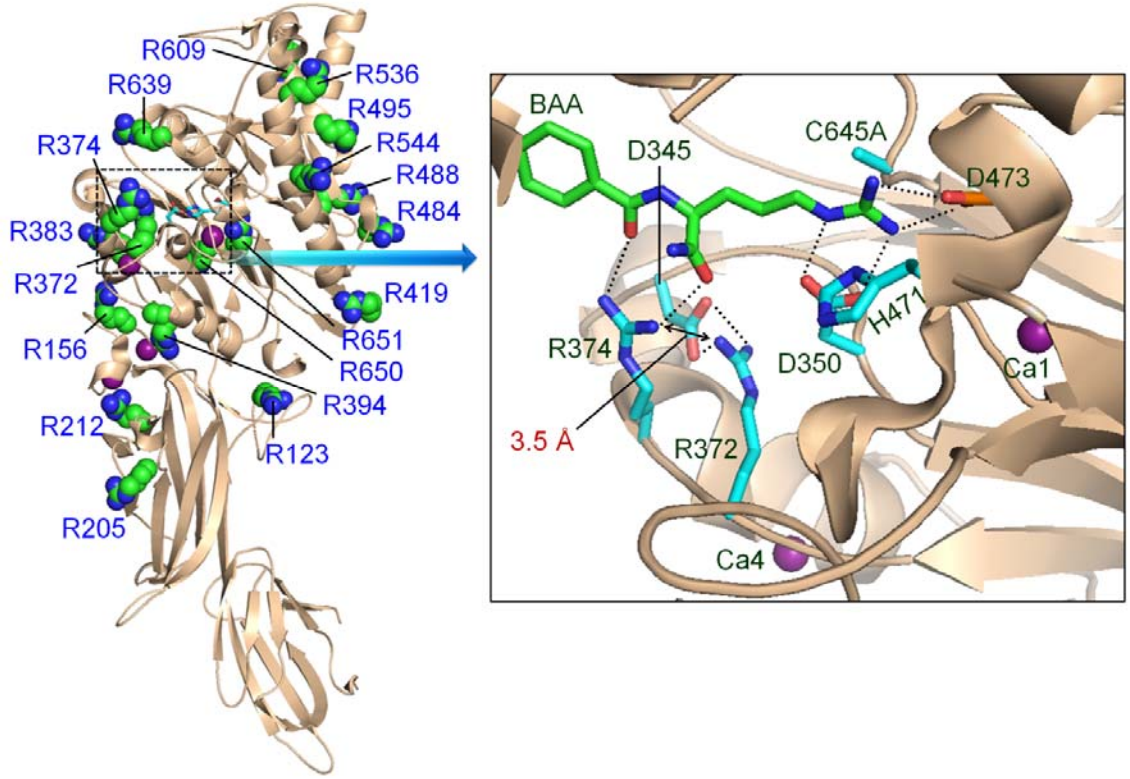

Supplementary Figure 11. Sites of PAD4 autocitrullination (Supplementary Table 1) (PDB code: 1WDA). R218 site could not be shown because of disorder in that region. While most of these sites are far from the active site and are on the surface of the protein, arginines 372 and 374 are close to the active site. Notably, the distance between these two positively-charged residues is only 3.5 Å, a value that is close to the sum of the N···N van der Waal's radii (3.1 Å). Electrostatic repulsions between R372 and R374 are delicately balanced by hydrogen bonding interactions with the substrate, BAA and an aspartate, D345. These observations suggest that the citrullination of either of these residues will likely perturb the delicate balance and affect enzymatic activity.

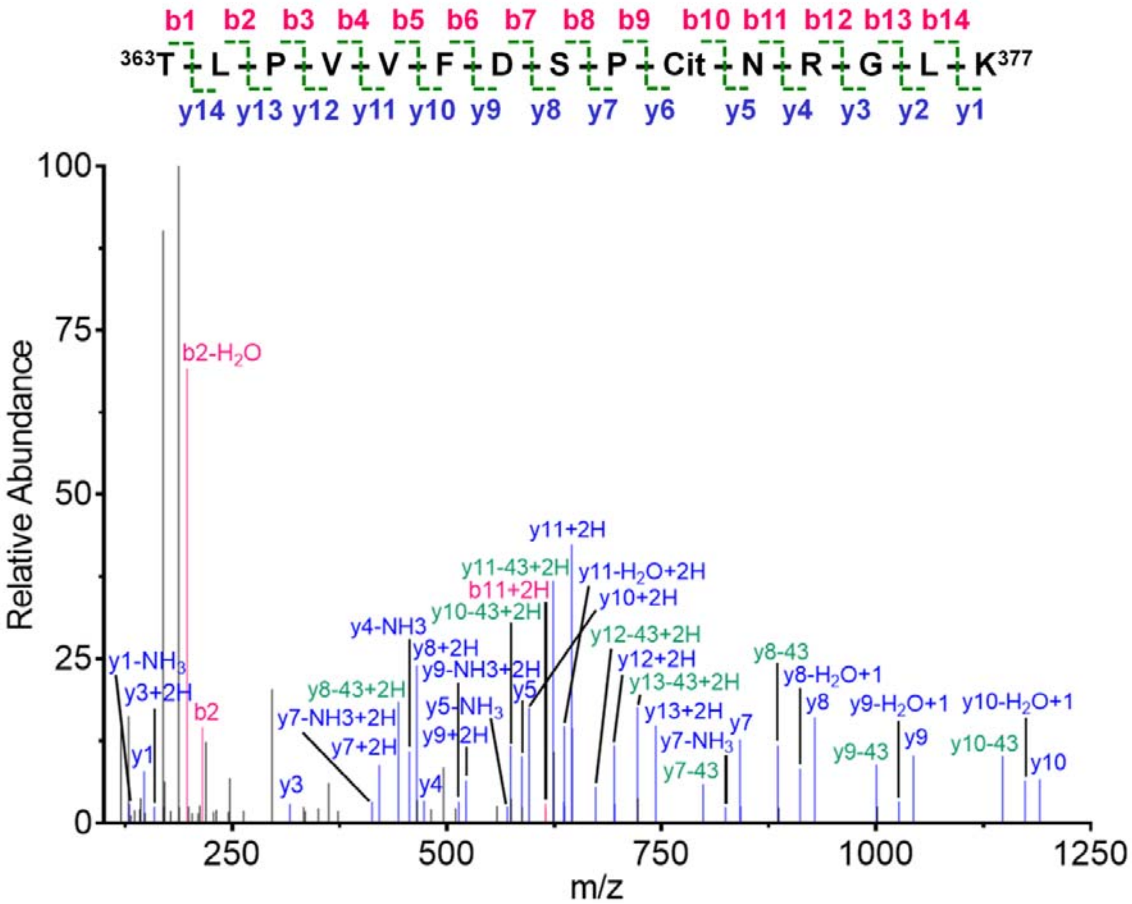

Supplementary Figure 12. MS2 spectrum of the peptide ([363]TLPVVFDSPCitNRGLK[377]) containing the Cit372 residue. This peptide was generated by digesting the R372Cit mutant PAD4 with Lys-C and Glu-C. A 43 Da neutral loss, characteristic to the citrulline side chain that loses isocyanic acid during fragmentation, from y7-y10, and not from y4-y5 ion confirms citrulline incorporation at the 372 position.

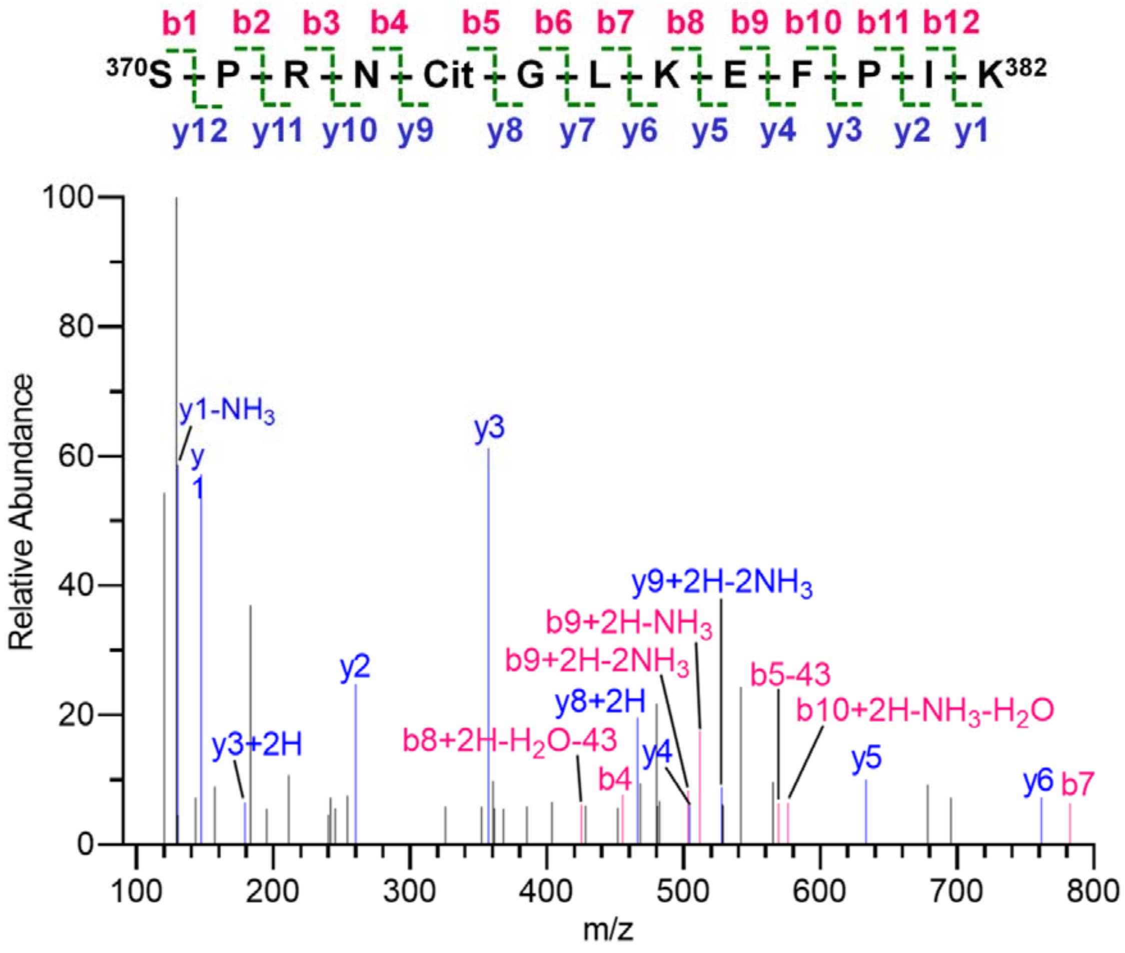

Supplementary Figure 13. MS2 spectrum of the peptide ([370]SPRNCitGLKEFPIK[382]) containing the Cit374 residue. This peptide was generated by digesting R374Cit mutant PAD4 with Lys-C and Glu-C. A 43 Da neutral loss, characteristic to the citrulline side chain that loses isocyanic acid during fragmentation, from b5, and not from b4 ion confirms citrulline incorporation at the 374 position.

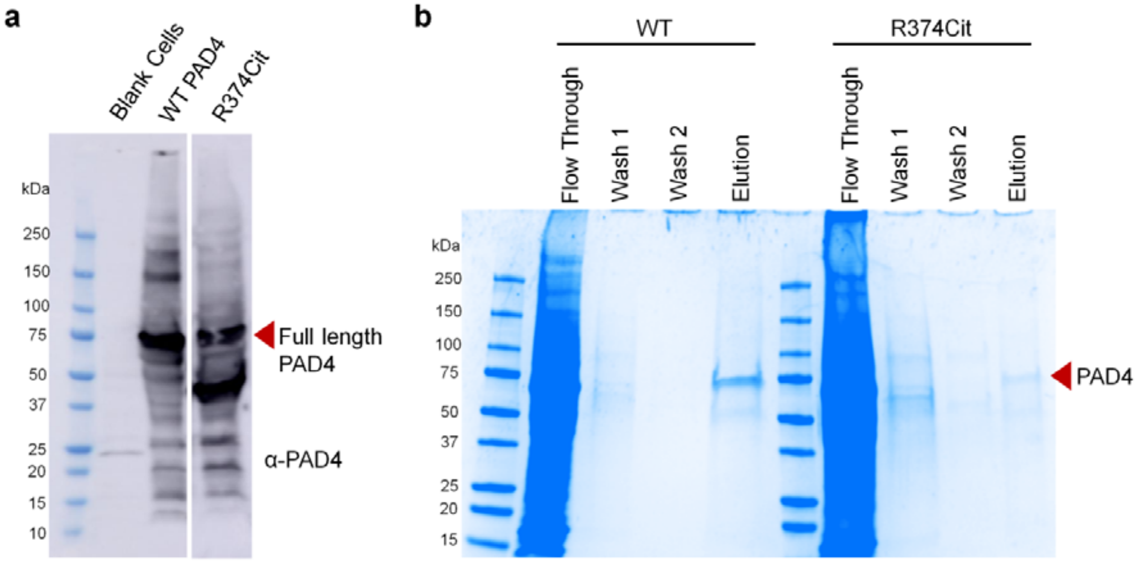

Supplementary Figure 14. **a**, Expression of WT and R374Cit PAD4 in EXPI293F cells as monitored by western blot analysis of the lysate using an α-PAD4 antibody. EXPI293F cell lysate (without transfection) served as a negative control. **b**, Coomassie stain of purified WT and R374Cit PAD4 from EXPI293F lysate by Ni-NTA affinity chromatography. Wash 1, Wash 2 and Elution buffer contained 50, 75 and 300 mM imidazole, and the elution fraction was dialyzed to remove the imidazole.

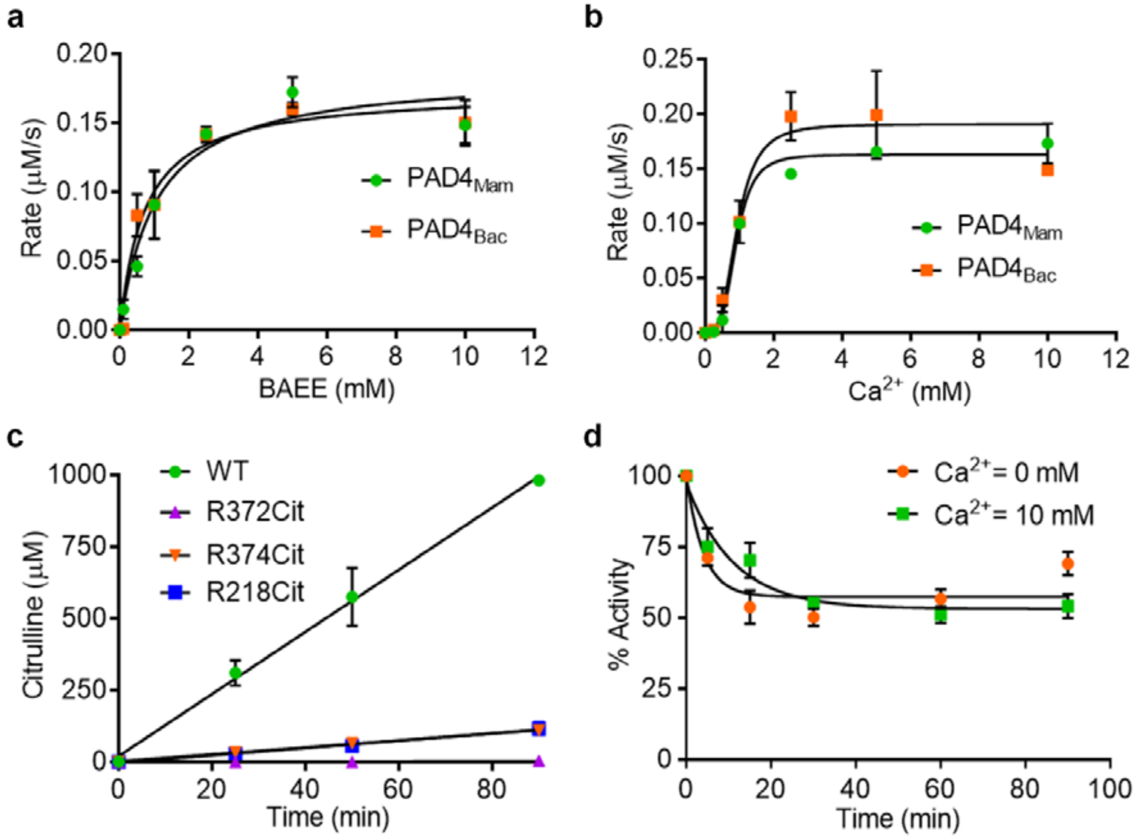

Supplementary Figure 15. **a**, Michaelis-Menten kinetics for the citrullination of BAEE by $PAD4_{Mam}$ (purified from mammalian expression system) and $PAD4_{Bac}$ (purified from bacterial expression system), indicating that both these enzymes have similar steady-state kinetic parameters (Supplementary Table 3). Since $PAD4_{Mam}$ contains an N-terminal FLAG and a C-terminal His tag, these results also indicate that the presence of these tags do not affect enzymatic activity. **b**, Calcium-dependence plots for $PAD4_{Mam}$ and $PAD4_{Bac}$. These data indicate that regardless of the source of the enzyme, the $K_{0.5}$ (Ca^{2+} concentration for half-maximal activity) values are 0.9 mM. **c**, Time-dependent citrulline production by WT, R372Cit and R374Cit PAD4. **d**, Effect of autocitrullination on the enzymatic activity of PAD4. The activity loss both in the presence and absence of calcium may be due to the oxidation of the active site cysteine over time. For all the panels, n=2 independent

experiments, data are presented as mean value ± SD. Source data for all panels are provided as a Source Data file.

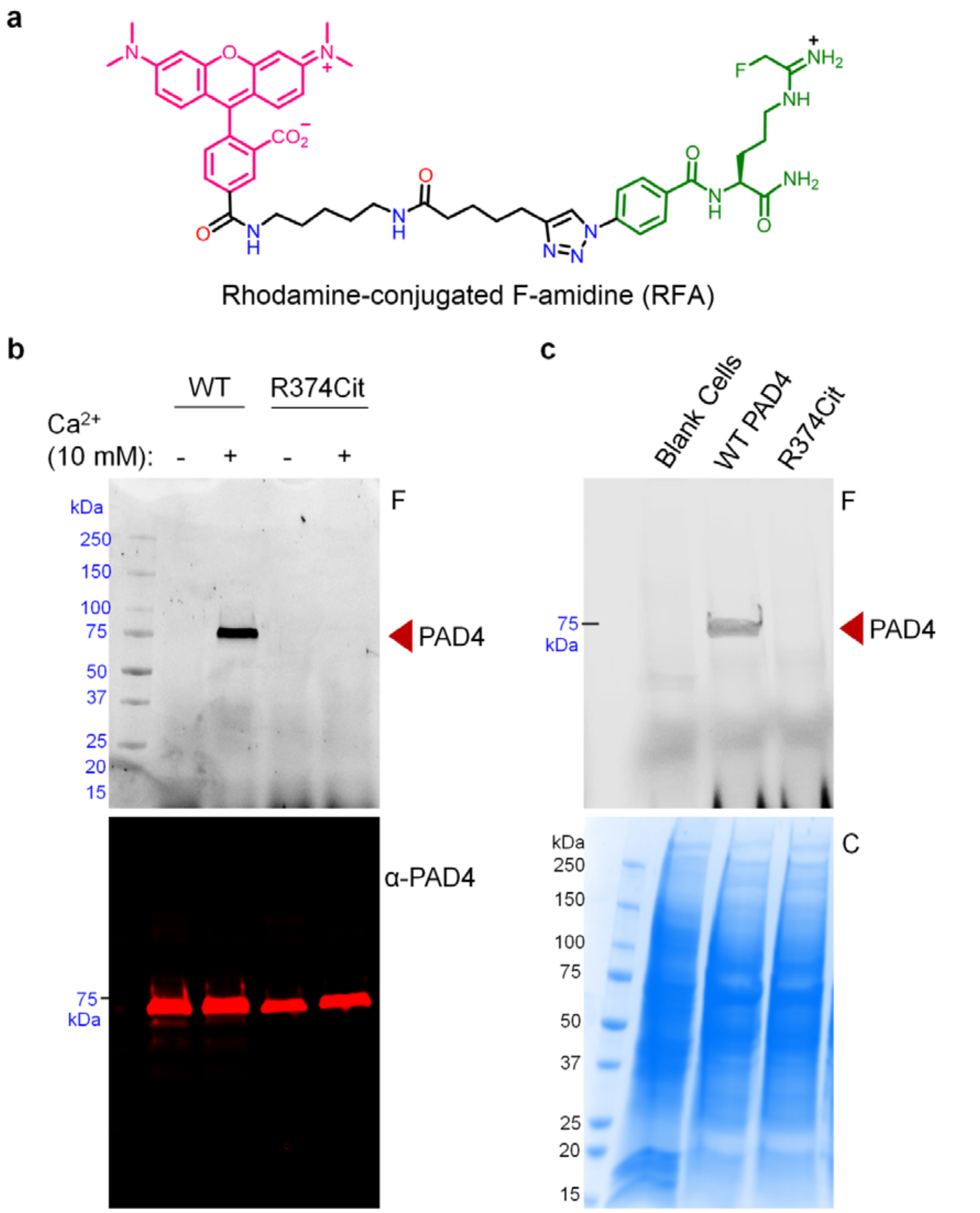

Supplementary Figure 16. **a**, Chemical structure of rhodamine-conjugated F-amidine (RFA). **b**, RFA-labeling of wild-type (WT) and R374Cit PAD4 in the presence and absence of Ca^{2+}. **c**, RFA-labeling of EXPI293F cell lysate containing wild-type (WT) and R374Cit PAD4. EXPI293F cell lysate (without transfection) served as a negative control. F and C refer to the fluorograph and Coomassie brilliant blue stain, respectively.

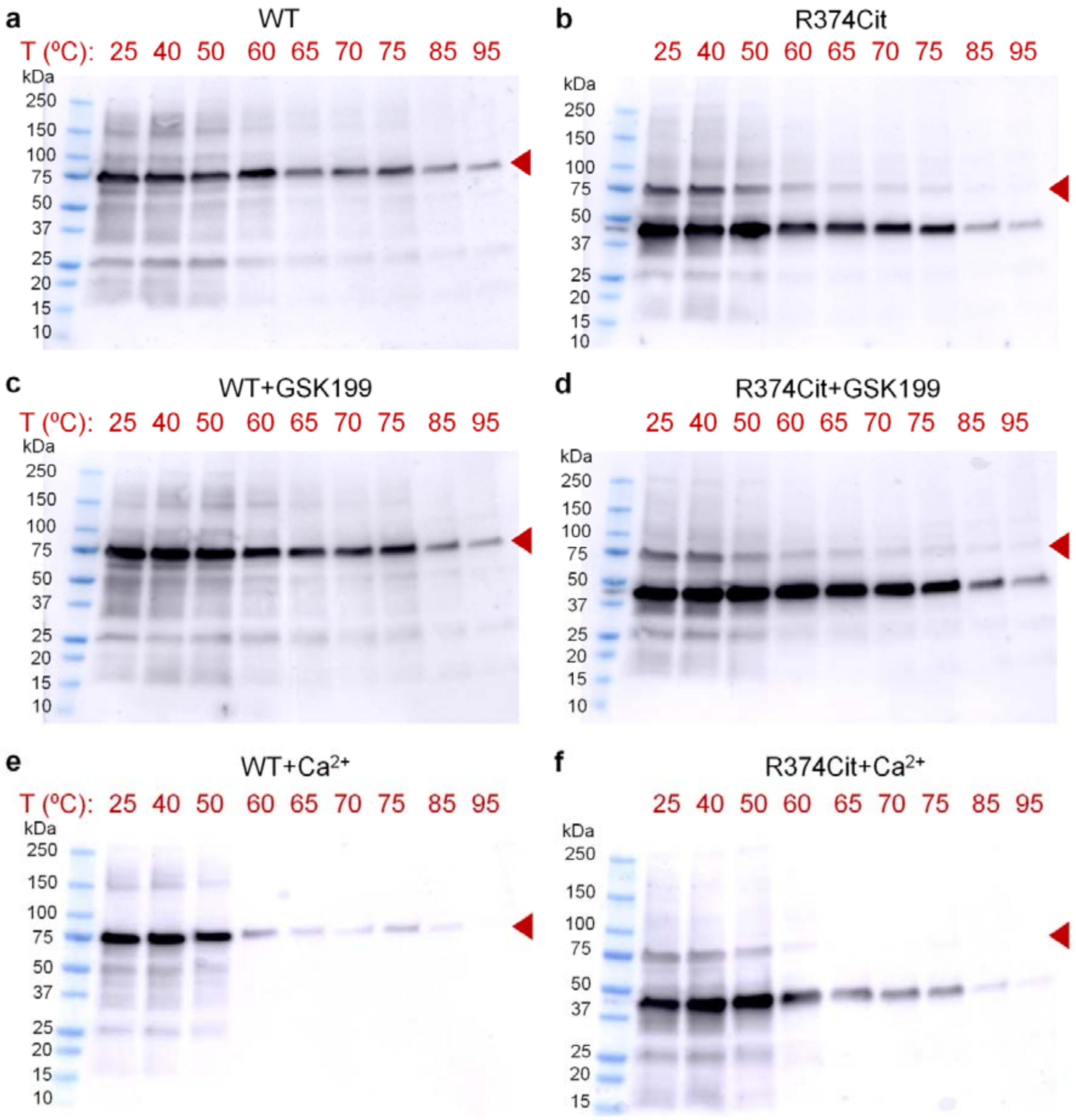

Supplementary Figure 17. Full western blot images for the thermal shift assays using EXPI293F cell lysate containing wild-type (WT) and R374Cit PAD4. **a**, WT PAD4. **b**, R374Cit PAD4. **c**, WT PAD4 + GSK199. **d**, R374Cit PAD4 + GSK199. **e**, WT PAD4 + Ca2^{+}. **f**, R374Cit PAD4 + Ca^{2+}. GSK199 and Ca^{2+} were used at 10 μM and 1 mM concentration, respectively. Full-length PAD4 is indicated by a brown triangle in each blot.

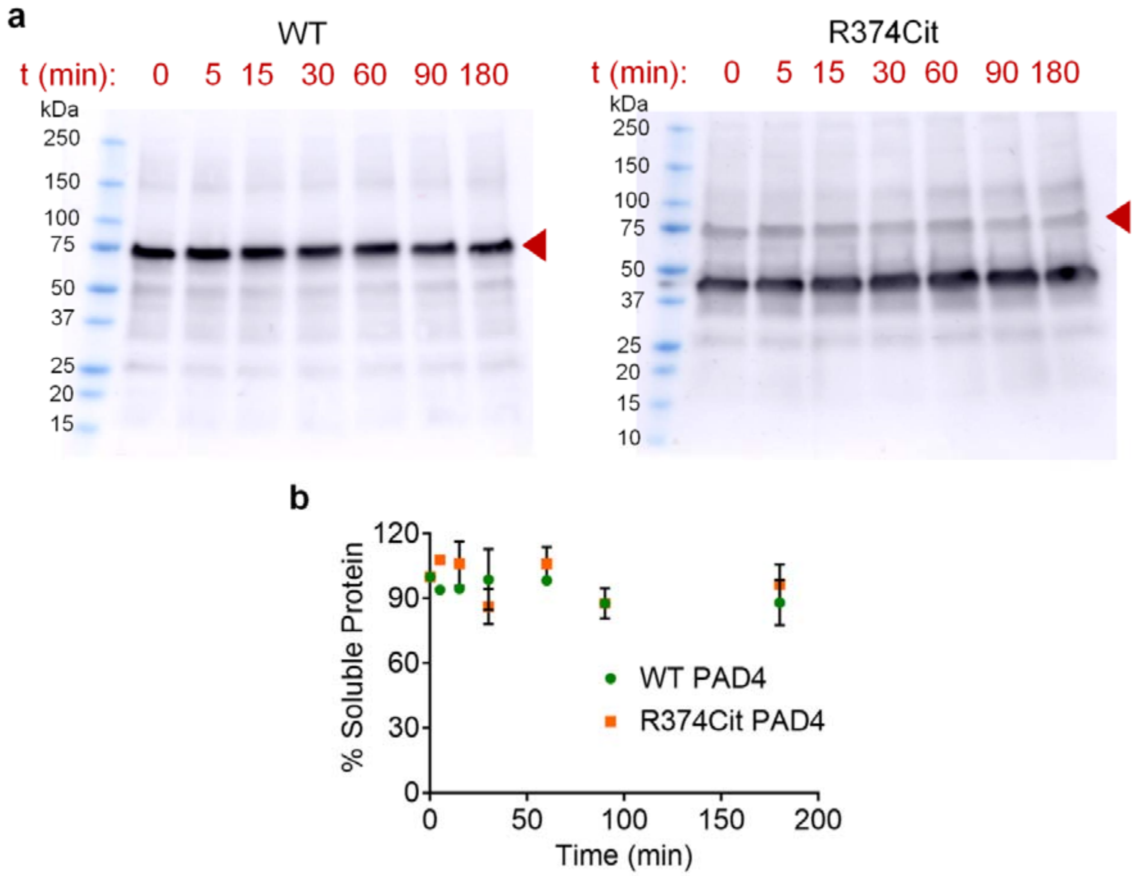

Supplementary Figure 18. **a**, Thermal stability of WT and R374Cit PAD4 at 37 ºC over 3 h. EXPI293F cell lysate containing WT PAD4 or R374Cit mutant was used in this assay. Full-length PAD4 is indicated by a brown triangle in each blot. **b**, Variation of PAD4 band intensities in panel **a** over 180 min, indicating that R374Cit mutant is as stable as WT PAD4 at 37 ºC. n=2 independent experiments, data are presented as mean value ± SD. Source data for panel **b** are provided as a Source Data file.

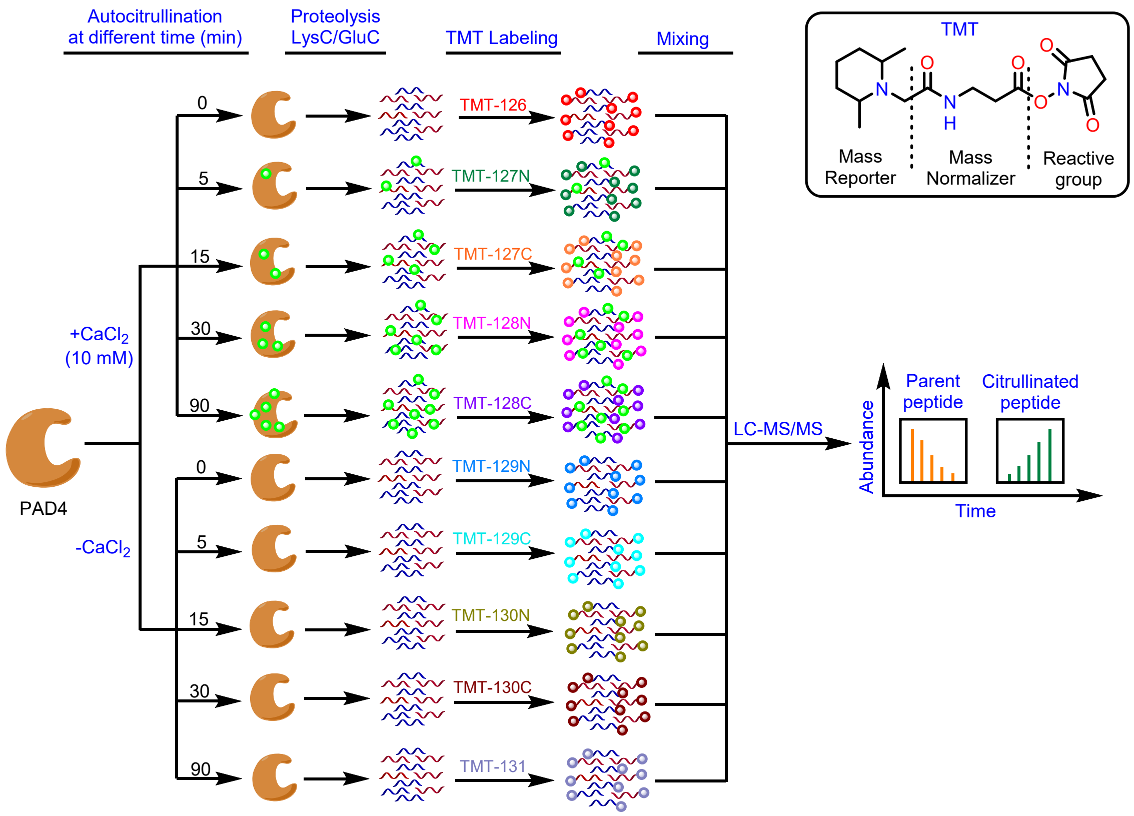

Supplementary Figure 19. Schematic representation of quantitative proteomic analysis of time-dependent autocitrullination of PAD4. Samples treated in the absence of calcium served as negative controls. Peptides derived from proteolysis of PAD4 with Lys-C and Glu-C were labeled with isobaric tandem mass tags (TMT) to enable simultaneous quantification of autocitrullination at various sites with increasing time.

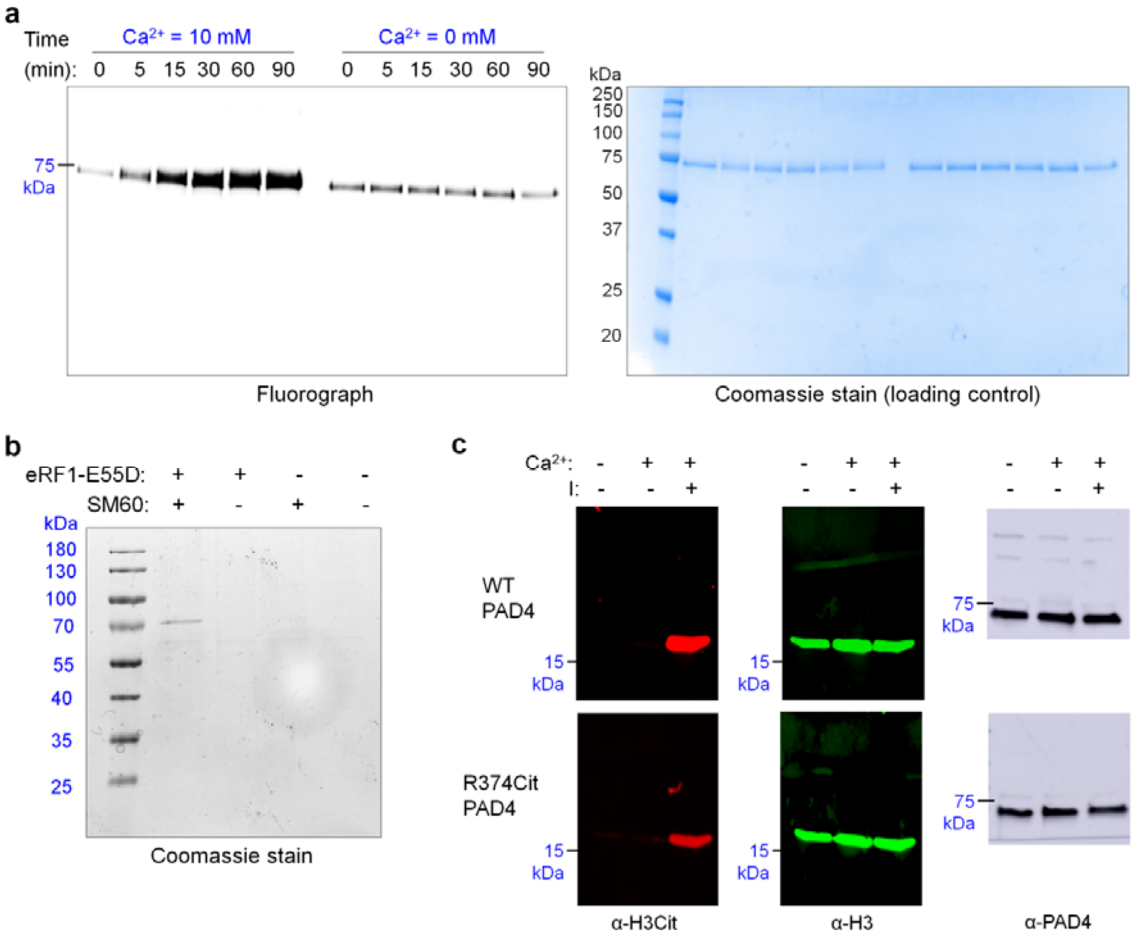

Supplementary Figure 20. Full gels corresponding to Fig. 3**a** (**a**), 3**b** (**b**) and 3**e** (**c**). The blots for histone H3 citrullination (**c**) by WT and R374Cit PAD4 were run separately. They were run separately because the extremely bright band due to citrullinated histone H3 obtained from WT PAD4 completely masks that from R374Cit PAD4 when they are run in the same gel.

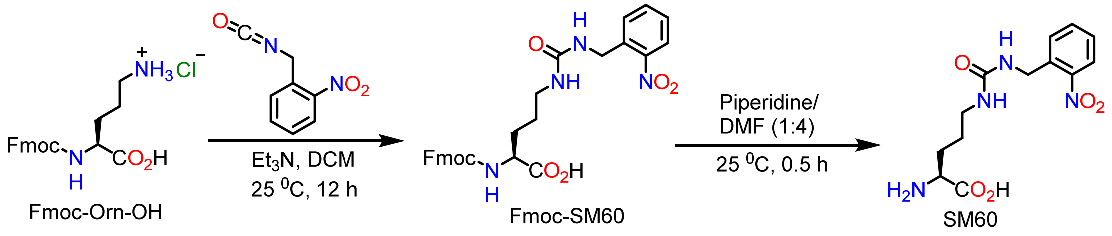

Supplementary Figure 21. Synthesis of SM60.

References

1. Slack, J.L., Jones, L.E., Jr., Bhatia, M.M. & Thompson, P.R. Autodeimination of protein arginine deiminase 4 alters protein-protein interactions but not activity. *Biochemistry* **50**, 3997-4010 (2011).
2. Andrade, F. *et al.* Autocitrullination of human peptidyl arginine deiminase type 4 regulates protein citrullination during cell activation. *Arthritis Rheum.* **62**, 1630-1640 (2010).
