# Supplementary material for: Site-specific incorporation of citrulline into proteins in mammalian cells
Source: Nat Commun. 2021 Jan 4;12:45. doi: 10.1038/s41467-020-20279-w (PMC7782748; doi:10.1038/s41467-020-20279-w)
Supplement: Supplementary file 2 — Reporting Summary [file 41467_2020_20279_MOESM2_ESM.pdf]

nature research

Corresponding author(s): Paul R Thompson
Abhishek Chatterjee

Last updated by author(s): Nov 12, 2020

Reporting Summary

Nature Research wishes to improve the reproducibility of the work that we publish. This form provides structure for consistency and transparency in reporting. For further information on Nature Research policies, see our Editorial Policies and the Editorial Policy Checklist.

Statistics

For all statistical analyses, confirm that the following items are present in the figure legend, table legend, main text, or Methods section.

n/a	Confirmed	
☐	☒	The exact sample size (n) for each experimental group/condition, given as a discrete number and unit of measurement
☐	☒	A statement on whether measurements were taken from distinct samples or whether the same sample was measured repeatedly
☒	☐	The statistical test(s) used AND whether they are one- or two-sided<br>*Only common tests should be described solely by name; describe more complex techniques in the Methods section.*
☒	☐	A description of all covariates tested
☐	☒	A description of any assumptions or corrections, such as tests of normality and adjustment for multiple comparisons
☐	☒	A full description of the statistical parameters including central tendency (e.g. means) or other basic estimates (e.g. regression coefficient) AND variation (e.g. standard deviation) or associated estimates of uncertainty (e.g. confidence intervals)
☒	☐	For null hypothesis testing, the test statistic (e.g. F, t, r) with confidence intervals, effect sizes, degrees of freedom and P value noted<br>*Give P values as exact values whenever suitable.*
☒	☐	For Bayesian analysis, information on the choice of priors and Markov chain Monte Carlo settings
☒	☐	For hierarchical and complex designs, identification of the appropriate level for tests and full reporting of outcomes
☒	☐	Estimates of effect sizes (e.g. Cohen's d, Pearson's r), indicating how they were calculated

Our web collection on statistics for biologists contains articles on many of the points above.

Software and code

Policy information about availability of computer code

Data collection	Topspin 3.6.2 (for NMR), ChemStation (Agilent Technologies, for LC-MS), Axiovision Rel 4.8 (for recording EGFP Fluorescence), SoftMax Pro 5.4 (for recording UV-VIS absorbance in microplate reader), EnVision Manager 1.14.3049.1193 (for recording fluorescence in microplate reader), Typhoon FLA 9000 version 1.2 (for in-gel fluorescence), ODYSSEY 3.0.30 (Licor, for Western blot images), ImageQuant TL 7.0 (for chemiluminescence), Image Lab 5.0 (for recording coomassie stains), Xcalibur 4.3 (for LC-MS/MS).
Data analysis	Graphpad Prism 8, Origin 9, ImageJ 1.52v, Microsoft excel 2010, Proteome discover 2.1, Mascot server 2.5, Scaffold 4.10.0

For manuscripts utilizing custom algorithms or software that are central to the research but not yet described in published literature, software must be made available to editors and reviewers. We strongly encourage code deposition in a community repository (e.g. GitHub). See the Nature Research guidelines for submitting code & software for further information.

Data

Policy information about availability of data

All manuscripts must include a data availability statement. This statement should provide the following information, where applicable:
- Accession codes, unique identifiers, or web links for publicly available datasets
- A list of figures that have associated raw data
- A description of any restrictions on data availability

All the raw data are available upon request to the corresponding authors by email. Source data for the relevant figures (1d, 2b, 3a, 3d-f, S10a-b, S15a-d, S18b) are provided with this paper.

Field-specific reporting

Please select the one below that is the best fit for your research. If you are not sure, read the appropriate sections before making your selection.

- [x] Life sciences
- [ ] Behavioural & social sciences
- [ ] Ecological, evolutionary & environmental sciences

For a reference copy of the document with all sections, see nature.com/documents/nr-reporting-summary-flat.pdf

Life sciences study design

All studies must disclose on these points even when the disclosure is negative.

Sample size	For all the in vitro experiments, the sample size is chosen to be 2 because these experiments afford highly reproducible results. Also, these experiments are traditionally performed with n=2 in the literature.<br>The sample size for all the in cellulo experiments is chosen to be 3 to get more reliability on the results.<br>No sample size calculation was performed.
Data exclusions	No data was excluded from the analyses.
Replication	Generally up to 10% standard deviation was allowed to consider a data as reproducible. All the attempts at replication were successful.
Randomization	This study does not involve any animal or human patient. Therefore, randomization was not necessary.
Blinding	All the experiments were performed in an unbiased way and the results were validated using multiple assays. Therefore, blinding was not necessary for this study.

Reporting for specific materials, systems and methods

We require information from authors about some types of materials, experimental systems and methods used in many studies. Here, indicate whether each material, system or method listed is relevant to your study. If you are not sure if a list item applies to your research, read the appropriate section before selecting a response.

Materials & experimental systems

n/a	Involved in the study	
[ ]	[x]	Antibodies
[ ]	[x]	Eukaryotic cell lines
[x]	[ ]	Palaeontology and archaeology
[x]	[ ]	Animals and other organisms
[x]	[ ]	Human research participants
[x]	[ ]	Clinical data
[x]	[ ]	Dual use research of concern

Methods

n/a	Involved in the study	
[x]	[ ]	ChIP-seq
[x]	[ ]	Flow cytometry
[x]	[ ]	MRI-based neuroimaging

Antibodies

Antibodies used	Anti-PAD4 (Abcam ab50332, polyclonal) (Dilution 1:1000)<br>Anti-Histone H3 (citrulline R2,8,17) (Abcam ab5103, polyclonal) (Dilution 1:1000)<br>Anti-Histone H3 (Abcam ab10799, monoclonal, clone number: mAbcam 10799) (Dilution 1:1000)
Validation	https://www.abcam.com/padi4--pad4-antibody-ab50332.html<br>https://www.abcam.com/histone-h3-citrulline-r2--r8--r17-antibody-ab5103.html<br>https://www.abcam.com/histone-h3-antibody-mabcam-10799-chip-grade-ab10799.html

Eukaryotic cell lines

Policy information about cell lines

Cell line source(s)	HEK293T (ATCC), EXPI293F (Gibco).<br>Rationale: EGFP and PAD4 are not found in these cell lines and therefore, they are suitable to overexpress EGFP and PAD4. Furthermore, the transfection efficiency is high in these cell lines.
Authentication	Cell lines obtained from manufacturer were used without further authentication.
Mycoplasma contamination	No Mycoplasma contamination was found.

Commonly misidentified lines<br>(See ICLAC register)	No commonly misidentified cell line was used in the study.
