# Supplementary material for: Site-specific incorporation of citrulline into proteins in mammalian cells
Source: Nat Commun. 2021 Jan 4;12:45. doi: 10.1038/s41467-020-20279-w (PMC7782748; doi:10.1038/s41467-020-20279-w)
Supplement: Supplementary file 3 — Peer Review File [file 41467_2020_20279_MOESM3_ESM.pdf]

Editorial Note: This manuscript has been previously reviewed at another journal that is not operating a transparent peer review scheme. This document only contains reviewer comments and rebuttal letters for versions considered at *Nature Communications*. Mentions of prior referee reports have been redacted.

Reviewers' Comments:

Reviewer #1:
Remarks to the Author:
The authors have addressed major comments from referees. The revised version is suitable for publication.

Reviewer #2:
Remarks to the Author:
The authors have clarified the questions and comments.
